# Use of Telehealth During the COVID-19 Pandemic: Scoping Review

**DOI:** 10.2196/24087

**Published:** 2020-12-01

**Authors:** Sathyanarayanan Doraiswamy, Amit Abraham, Ravinder Mamtani, Sohaila Cheema

**Affiliations:** 1 Institute for Population Health Weill Cornell Medicine Doha Qatar

**Keywords:** COVID-19, telehealth, telemedicine, scoping review

## Abstract

**Background:**

With over 37.8 million cases and over 1 million deaths worldwide, the COVID-19 pandemic has created a societal and economic upheaval of unparalleled magnitude. A positive transformation has been brought about by innovative solutions in the health care sector that aim to mitigate the impact of COVID-19 on human health. For instance, the use of telehealth has been on the rise amidst this public health emergency.

**Objective:**

Given the unprecedented scale of the pandemic with no definitive endpoint, we aimed to scope the existing telehealth-related literature during a defined period of the ongoing pandemic (ie, January to June 2020).

**Methods:**

Our scoping review was guided by the Joanna Briggs Institute Reviewer Manual. We systematically searched PubMed and Embase databases with specific eligibility criteria. Data extracted from the shortlisted articles included first author and affiliation, journal title, publication type, terminologies used to describe telehealth and their accompanying definitions, health discipline or medical specialties and subspecialties wherein telehealth had been applied, the purpose of telehealth use, and the authors’ overall sentiment on telehealth use. We collated the available information and used descriptive statistics to analyze the synthesized data.

**Results:**

In all, 543 articles published across 331 different journals were included in this scoping review. The Journal of Medical Internet Research and its sister journals featured the highest number of articles (25/543, 4.6%). Nearly all (533/543, 98.2%) articles were in English. The majority of the articles were opinions, commentaries, and perspectives (333/543, 61.3%). Most authors of the articles reviewed were from high-income countries (470/543, 86.6%), especially from the United States of America (237/543, 43.6%). In all, 39 different definitions were used to describe terms equivalent to telehealth. A small percentage (42/543, 7.7%) of the articles focused on the provision of COVID-19–related care. Moreover, 49.7% (270/543) of the articles primarily focused on the provision of multiple components of clinical care, and 23% (125/543) of the articles focused on various specialties and subspecialties of internal medicine. For a vast majority (461/543, 84.9%) of the articles, the authors expressed a celebratory sentiment about the use of telehealth.

**Conclusions:**

This review identified considerable emerging literature on telehealth during the first six months of the COVID-19 pandemic, albeit mostly from high-income countries. There is compelling evidence to suggest that telehealth may have a significant effect on advancing health care in the future. However, the feasibility and application of telehealth in resource-limited settings and low- and middle-income countries must be established to avail its potential and transform health care for the world’s population. Given the rapidity with which telehealth is advancing, a global consensus on definitions, boundaries, protocols, monitoring, evaluation, and data privacy is urgently needed.

## Introduction

The ongoing COVID-19 pandemic is a defining moment in the 21^st^ century for many reasons. It has affected over 18.3 million people worldwide and led to over 695,000 deaths, resulting in a societal and economic upheaval of unparalleled magnitude [1]. These unprecedented times have also highlighted the power of science in identifying creative solutions to address this mammoth global challenge. Numerous Information and Communication technology (ICT) tools and innovative approaches, such as tools for online education and telecommuting, were being developed even before the pandemic; these tools gained popularity as people sought to find creative solutions to mitigate the impact of the pandemic [2]. In the health care sector, telehealth or telemedicine practices expanded tremendously during the pandemic and continue to flourish [3].

Telemedicine, a term coined in the 1970s, meant “healing at a distance” [4]. Over the following 4 decades, several peer-reviewed definitions for the term have emerged. In 2007, the World Health Organization (WHO) introduced a standardized definition for telemedicine: “The delivery of healthcare services, where distance is a critical factor, by all healthcare professionals using information and communication technologies for the exchange of valid information for diagnosis, treatment and prevention of disease and injuries, research and evaluation, and for the continuing education of healthcare providers, all in the interests of advancing the health of individuals and their communities” [5]. ICT is defined as a “diverse set of technological tools and resources used to transmit, store, create, share or exchange information. These technological tools and resources include computers, the Internet (websites, blogs, and emails), live broadcasting technologies (radio, television, and webcasting), recorded broadcasting technologies (podcasting, audio and video players and storage devices), and telephony (fixed or mobile, satellite, visio/video-conferencing, etc)” [6].

Although used interchangeably, telehealth by definition refers to health care services involving all health care professions (including education of health care professionals themselves), whereas telemedicine refers to services delivered by physicians only [7]. The last 2 decades have seen the emergence of newer terms such as ehealth, mobile health (mhealth), and digital health, to accommodate more recent advances in ICT-enabled health care [8]. For consistency, and as population health researchers with an interest in the broader health care domain beyond the only physician-delivered medical care model, in this paper, we prefer to use the terminology “telehealth” to refer to all forms of ICT-enabled health care.

Global interest within the scientific community for using telehealth was on the rise even before the COVID-19 pandemic, as evidenced by the increasing number of studies published on this topic in recent years [9]. However, the use of telehealth to improve patient care and population health has been predominantly concentrated among high-income countries rather than low- and low-middle–income countries [10]. Some medical specialties, such as radiology, dermatology, pathology, and psychiatry, used telehealth more frequently than others [7]. Health care professionals express varying sentiments regarding the use of telehealth for patient care. Some consider telehealth as the new holy grail in health care, whereas others are guarded in their opinion about its applicability in the field. Some worry about the lack of face-to-face connection between patients and health care providers, which they believe is needed to develop a therapeutic bond, and others express concern that clinicians are unable to perform all aspects of physical examination while using telehealth [11]. The risk of widening inequity across various population subgroups with the advent of telehealth is also a prevailing concern [12].

With the advancement of ICT in the health care sector to ensure accountability, ethical medical practice, and patient data privacy, several countries imposed legal restrictions and strict regulations regarding the use of this rapidly expanding technology. These factors, in addition to the lack of insurance coverage, were reported as primary barriers to advancing telehealth in many countries prior to the COVID-19 pandemic [13]. With the onset of the pandemic, clinics and hospitals rapidly restricted access for emergency care in order to reduce the risk of disease transmission. To manage patient load, safeguard the health of patients and health care professionals, and ensure continuity of patient care, capable health care systems expanded their health care delivery by providing telehealth services. Moreover, several countries relaxed their laws and regulations pertaining to the use of telehealth. Additionally, with the evolving landscape of health care delivery during the COVID-19 pandemic, insurance companies have now started reimbursing expenses for patient care delivered via telehealth [13].

We hypothesized that there has likely been an increase in the delivery of telehealth-enabled care since the onset of the COVID-19 pandemic, with concurrent experiential reporting by health care professionals who are using telehealth as a health care delivery modality. The application of telehealth to promote health, evaluate and manage diseases, and rehabilitate individuals has been documented in other public health emergencies [14]. Given the unprecedented scale of what we are witnessing in the current COVID-19 pandemic, and its surrounding uncertainties and ramifications on future health care delivery, we aimed to scope the existing literature on telehealth during a defined period in the ongoing pandemic (ie, January to June 2020).

## Methods

### Study Design

We performed a scoping review consistent with the guidance provided by the Joanna Briggs Institute Reviewer Manual [15,16]. The scoping review follows the PRISMA-ScR (Preferred Reporting Items for Systematic Reviews and Meta-Analyses Extension for Scoping Reviews) checklist (see Multimedia Appendix 1) [17]. The protocol was registered on the Open Science Framework (registration DOI: 10.17605/OSF.IO/AXN32) [18] on July 19, 2020.

### Eligibility Criteria

The eligibility criteria were established a priori. We considered only publications that had been accepted for publication or had been published in peer-reviewed journals. Preprints were not considered. All publication types were considered, including opinions, viewpoints, original research articles, and reviews, with no geographic, time, or language restrictions. Furthermore, we included all publications examining any aspect of telehealth from direct, synchronous videoconferencing between patients and health care providers, to mhealth monitoring via apps, as well as wearable smart devices. We excluded any article whose primary focus was not telehealth, including articles related to molecular studies, modeling studies, and studies that used technology only for a better understanding of disease dynamics with no immediate and direct benefit for health care workers (including medical students and health care managers) or patients.

### Search Strategy

We systematically searched 2 electronic databases (PubMed and Embase) from January 1 to June 10, 2020, using both keywords and controlled vocabulary (such as MeSH terms). The search terms were a combination of 2 concepts: (1) COVID-19 and (2) telehealth or telemedicine. The detailed search strategy is provided in Multimedia Appendix 2*.* A senior information specialist validated the search strategy. For a comprehensive assessment, we also searched the reference lists of all the included articles to identify other studies that may be relevant to our review.

### Article Selection and Data Extraction

Articles identified by our search strategy were imported into Rayyan, the online systematic review software, and duplicates were removed [19]. SD screened the title and abstracts of the identified articles. AA checked the excluded studies and was able to confirm that the exclusion criteria were correctly applied. Subsequently, SD and AA individually extracted data from 50% of the included studies each. We developed a standardized Microsoft Excel (Microsoft Corp.) template for data extraction to tabulate specific information from the included studies, such as journal title, written language of the article, reference to telehealth or its variants in the article title, reference to COVID-19 or its variants in the article title, publication type, country of the first author’s affiliation, country of focus of the article, terminologies used to describe telehealth and their accompanying definitions, the purpose of telehealth use, and the health discipline or medical specialty and subspecialties wherein telehealth had been applied during the study period.

Additionally, for each article, we categorized the overall sentiment expressed by the authors about the usefulness of telehealth. This categorization was based on the framework developed by Nettleton et al [20] and subsequently used by Dol et al [21] in their scoping reviews of the use of internet technology and social media in medical, sociological, and popular literature. Accordingly, the sentiments were categorized as “celebratory” (ie, authors provide a positive appraisal of telehealth use during the pandemic), “contingent” (ie, authors recognize the potential positive contribution but also acknowledge its potential limitations), or “concerned” (ie, authors identify challenges and caution on the imbalance that telehealth may create in health care delivery).

Finally, SD and AA randomly cross-checked approximately 10% of each other’s extractions to ensure correctness and completeness of the extracted data. No discrepancies were noted.

### Data Analysis and Synthesis

We synthesized the collated data by using descriptive statistics (frequencies and proportions). We used Microsoft Excel and SPSS software (version 26.0; IBM Corp) to analyze the data.

## Results

### Selection of Articles

**Figure 1:**
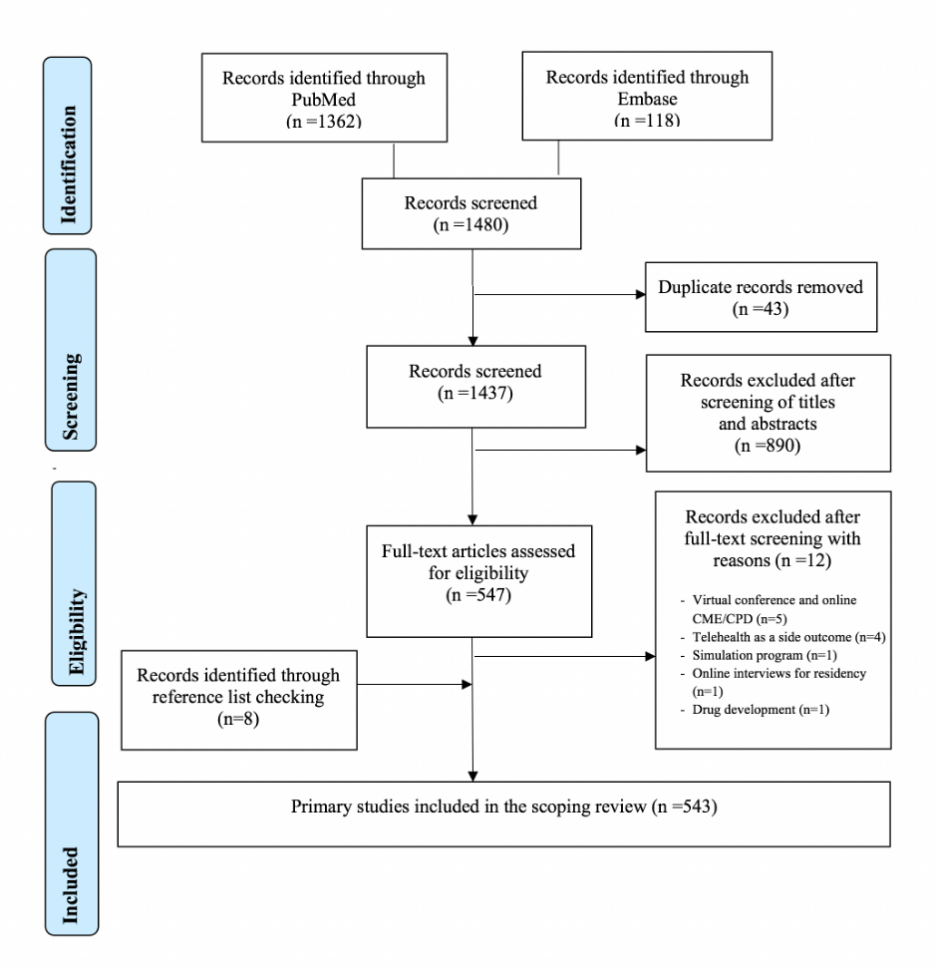
PRISMA 2009 flowchart. PRISMA: Preferred Reporting Items for Systematic Reviews and Meta-Analyses. CME: continuing medical education. CPD: continuing professional development.

After removal of duplicates, we identified 1437 articles in our initial search. Next, based on our eligibility criteria, articles were excluded at various screening stages: 890 at the title and abstract screening stage and 12 at the full-text screening stage. We also searched the reference lists of the included articles and found another 8 relevant articles for inclusion. Thus, a total of 543 articles were included in our review. Figure 1 shows the PRISMA flow chart illustrating the publication selection process. The full list of included studies is provided as Multimedia Appendix 3.

### Characteristics of Included Articles

The 543 articles included in our review were published across 331 journals. The *Journal of Medical Internet Research* and its sister journals featured the highest number of articles (25/543, 4.6%), followed by *Telemedicine and e-Health* (19/543, 3.5%). Other journals featuring a high number of included articles were the *Journal of Diabetes Science and Technology* (18/543, 3.3%), *Journal of the American Academy of Dermatology* (9/543, 1.7%), *Otolaryngology-Head and Neck Surgery* (9/543, 1.7%), and the *Journal of Rural Health* (8/543, 1.5%). The remaining journals featured fewer than 8 articles on telehealth in the context of COVID-19. Nearly all (533/543, 98.2%) of the included articles were in English. A small number of articles (8/543, 1.5%) were in French and Spanish, and 1 article each was in German and Hungarian. While the authors of this study read English and French, information from the Spanish, German, and Hungarian articles was extracted from their English abstracts by using Google Translate.

Our manual search of only the titles of the articles included in our study identified that 72.4% (393/543) of the articles made a direct reference to telehealth or its variants and 95.6% (519/543) of them made a direct reference to COVID-19 or its variants. A majority (333/543, 61.3%) of the articles were published as an opinion, commentary, and perspective, followed by empirical research (63/543, 11.6%) and review (narratives or systematic review and meta-analyses: 33/543, 6.1%). In all, 5% (27/543) of the articles were categorized as “others” (ie, Bridging the gap, Business horizons, Care delivery, Clinical forum, Clinical practice guidelines, Consensus statement, Curb side consult, First view, How to do it, Leaders focus, Orthopedic forum, Practice, Practice guidelines, Practice & policy, Special feature, and Training room). Article-type categorization was unclear for 16% (87/543) of the articles.

**Figure 2:**
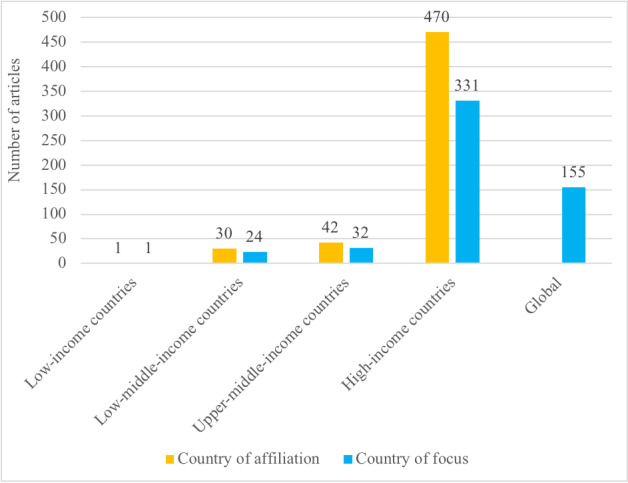
Number of articles based on the World Bank’s classification of countries by income level (N=543).

**Figure 3:**
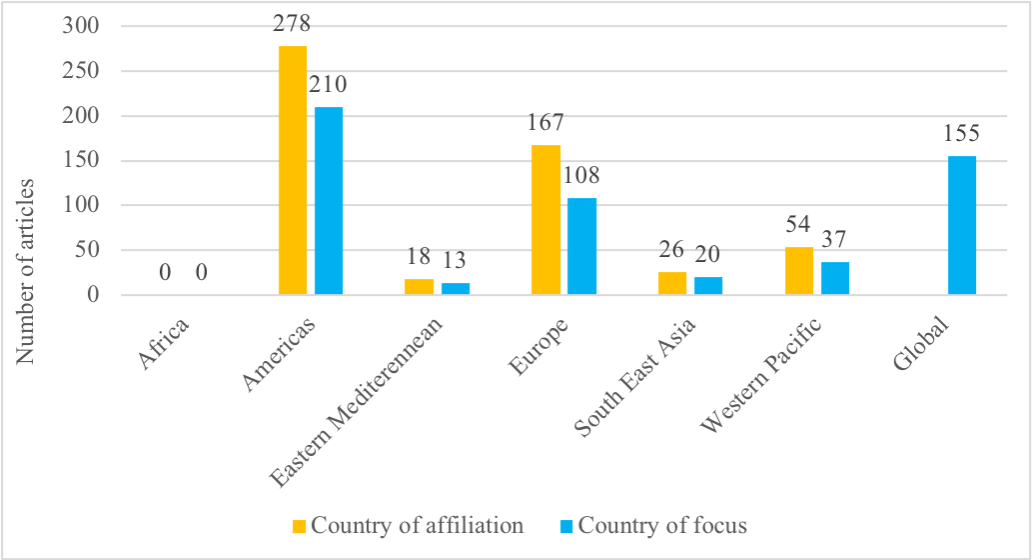
Number of articles based on the World Health Organization’s regional classification (N=543).

The first authors of the articles included in our review were affiliated (place of work) in 42 different countries. Most first authors were from the United States of America (237/543, 43.6%), followed by much a smaller number from the United Kingdom, Italy, India, Canada, Australia, France, China, Spain, and Singapore, in decreasing order (Table 1). The geographic focus of the articles also varied, with 32.8% (178/543) of all articles focusing on the United States; this was closely followed by 28.5% (155/543) of all articles had a global or regional focus (Table 2). The geographical focus of the articles largely matched the countries of affiliations of the first authors*.* We further grouped the countries according to the World Bank’s income status [22] and WHO’s regional classification [23]. The complete lists of countries based on the above classifications are provided in Multimedia Appendix 4. The vast majority of articles were published from high-income countries (470/543, 86.6%; World Bank classification) and the Americas region (277/543, 51%; WHO classification), closely followed by Europe (168/543, 30.9%). A summary of this comparison is provided in Figures 2 and 3.

**Table 1:** Country of affiliation of the first authors (N=543).

Country of affiliation^a^	Number of articles, n (%)
USA	237 (43.6)
UK	52 (9.6)
Italy	44 (8.1)
India	25 (4.6)
Canada	25 (4.6)
Australia	16 (2.9)
France	15 (2.8)
China	15 (2.8)
Spain	13 (2.4)
Singapore	11 (2.0)

^a^The first author’s place of work

**Table 2:** Geographic focus of published articles included in the review (N=543).

Country of focus^a^	Number of articles, n (%)
USA	178 (32.8)
Global^b^	155 (28.5)
UK	33 (6.1)
Italy	31 (5.7)
India	19 (3.5)
Canada	18 (3.3)
France	12 (2.2)
Australia	11 (2.0)
China	11 (2.0)
Spain	9 (1.7)
Brazil	9 (1.7)
Iran	6 (1.1)
Germany	6 (1.1)

^a^Some articles focused on more than one country.

^b^Articles that had a global focus, covering more than 3 countries.

### Terminologies Used and Their Definitions

We found 39 different terminologies related to telehealth in the articles included in our review. The most frequent terminologies used were “telehealth” and “telemedicine.” Additionally, frequent use of tele-prefix specialty or subspecialty (eg, teleneurology, telestroke), digital health, ehealth, remote health, video visits, and others was observed. Articles also included references to emerging ICT concepts such as artificial intelligence (AI), robotics, and wearable technology in the context of telehealth. Of the 543 articles, 105 (19.3%) articles included a definition of telehealth-related terminologies. Of these, 13 articles attempted to define telehealth, whereas 52 articles used varying definitions for telemedicine, only 2 of which matched the definition put forth by the WHO. The various definitions of “telehealth” and its variants provided in the included articles have been compiled and presented in Multimedia Appendix 5*.*

### Purpose of Telehealth Use

Of the articles included in our review, 7.7% (42/543) articles focused on the provision of telehealth related to COVID-19, whereas 92.3% (501/543) reported provision of health care support for conditions not related to COVID-19 (eg, tuberculosis, HIV, diabetes, and stroke). We found that the actual purpose of telehealth use during the COVID-19 pandemic varied across studies (Table 3), with the most common purpose being providing multiple components of clinical care (270/543, 49.7%), including any combination of triage, diagnosis, treatment, follow-up, and rehabilitation services. Other purposes for telehealth use were follow-up care (83/543, 15.3%), medical education (54/543, 9.9%), diagnosis only (39/543, 7.2%), and rehabilitation (24/543, 4.4%).

**Table 3:** Various purposes of telehealth use during the COVID-19 pandemic (N=543).

Purpose	Number of articles, n (%)
Clinical care	270 (49.7)
Follow-up	83 (15.3)
Medical education	54 (9.9)
Diagnosis	39 (7.2)
Rehabilitation	24 (4.4)
Health communication	20 (3.7)
Triage	19 (3.5)
Surveillance or contact tracing	16 (2.9)
Research	12 (2.2)
Health care worker wellbeing	6 (1.1)

### Telehealth Use in Various Medical Specialties and Subspecialties

With regard to the specialties covered in the articles included in our review, 89.9% (488/543) of the articles discussed the application of telehealth in medicine and dentistry, and 9.9% (54/543) of the articles focused on medical education. Among the 54 articles on medical education, 10 articles discussed residency training, 8 discussed undergraduate medical student training, and the remaining 36 discussed telehealth in the context of medical education in general (covering undergraduate, postgraduate, residency training, and continuing professional development programs). Only 1 article discussed the application of telehealth in dentistry. We further classified the specialties and subspecialties of medicine covered by the articles using the framework proposed by the American Association of Medical Colleges on various specialties and subspecialties of medicine [24]. We found that 12.9% (70/543) of the articles focused on the use of telehealth for medicine in general with no reference to any specialty. The majority of the remaining articles focused on telehealth use in the following medical specialties: internal medicine (125/543, 23%), preventive medicine (56/543, 10.3%), psychiatry (42/543, 7.7%), surgery (36/543, 6.6%), neurology (33/543, 6.1%), otolaryngology (23/543, 4.2%), and dermatology (23/543, 4.2%). Additional analysis of the subspecialties revealed that the top 5 subspecialties deploying telehealth were endocrinology (30/543, 5.5%), oncology (25/543, 4.6%), geriatrics (23/543, 4.2%), cardiovascular (20/543, 3.7%), and orthopedics (10/543,1.8%). The numbers of articles grouped across various specialties and subspecialties of medicine are reported in Table 4*.*

**Table 4:** Number of articles included in the review categorized according to various medical specialties, subspecialties, and specific disease conditions (N=543).

Medical specialty^a^, subspecialty^b^, and specific disease or condition^c^			Number of articles, n (%)
**Internal medicine**			125 (23)
	**Endocrinology**		30 (5.5)
		Diabetes mellitus/gestational diabetes	9 (1.7)
		Diabetes insipidus	1 (0.2)
		Hyponatremia	1 (0.2)
		Thyroid conditions	1 (0.2)
		Eating disorders	1 (0.2)
	**Oncology**		25 (4.6)
		Head and neck	8 (1.5)
		Lung	3 (0.6)
		Neurosurgical oncology	2 (0.4)
		Radiation oncology	2 (0.4)
		Surgical oncology	1 (0.2)
		Myelofibrosis	1 (0.2)
		Melanoma	1 (0.2)
		Prostate and other urological cancers	1 (0.2)
		Cardio-oncology	1 (0.2)
	**Geriatrics**		23 (4.2)
		Physical activity	1 (0.2)
	**Cardiovascular**		20 (3.7)
		Heart failure	4 (0.7)
		Aortic stenosis	1 (0.2)
		Vascular	1 (0.2)
	**Gastroenterology**		9 (1.7)
		Inflammatory bowel disease	5 (0.9)
		Hepatology	4 (0.7)
	**Rheumatology**		9 (1.7)
		Systemic lupus erythematosus	2 (0.4)
		Systemic sclerosis	1 (0.2)
		Rheumatoid arthritis	1 (0.2)
	**Pulmonology**		4 (0.7)
		Cystic fibrosis	2 (0.4)
		Asthma	1 (0.2)
		Severe respiratory failure (ECMO^d^)	1 (0.2)
	Critical care		3 (0.6)
	**Hematology**		2 (0.4)
		Hemophilia	2 (0.4)
No particular specialty mentioned^e^			70 (12.9)
**Psychiatry**			42 (7.7)
	Substance use		4 (0.7)
	Counselling		4 (0.7)
	Mood disorders		1 (0.2)
	Eating disorders		1 (0.2)
**Preventive medicine**			56 (10.3)
	COVID-19		42 (7.7)
	Health education		1 (0.2)
	Noncommunicable diseases		1 (0.2)
**Surgery**			36 (6.6)
	Orthopedics		10 (1.8)
	Neurosurgery		8 (1.5)
	Transplant		3 (0.6)
	Orofacial		2 (0.4)
	Trauma		1 (0.2)
	Thoracic		1 (0.2)
	Plastic		1 (0.2)
**Neurology**			33 (6.1)
	Epilepsy		5 (0.9)
	Stroke		4 (0.7)
	Amyotrophic lateral sclerosis/motor neuron disease		3 (0.6)
	Parkinson’s disease and movement disorders		3 (0.6)
	Migraine		2 (0.4)
	Dementia		2 (0.4)
	Multiple sclerosis		1 (0.2)
	Demyelinating diseases		1 (0.2)
**Otolaryngology**			23 (4.2)
	Pediatric ENT		2 (0.4)
	Dysphagia and swallowing disorders		2 (0.4)
	Speech pathology/laryngology		1 (0.2)
	Speech apnea		1 (0.2)
**Dermatology**			22 (4.1)
	Psoriasis		1 (0.2)
	Dermatosis		1 (0.2)
	Chronic inflammatory skin diseases		1 (0.2)
	Atopic dermatitis		1 (0.2)
	Cutaneous lesions		1 (0.2)
	Acne		1 (0.2)
**Pediatrics**			15 (2.8)
	Gastroenterology		3 (0.6)
	Neonatology		2 (0.4)
	Well-baby clinic		1 (0.2)
	Neurology		1 (0.2)
	Rehabilitation		1 (0.2)
	Inherited metabolic diseases		1 (0.2)
	Cleft palate/lip		1 (0.2)
	Overweight/obese children		1 (0.2)
	Adolescent health/eating disorders		1 (0.2)
	Adolescent health/ADHD		1 (0.2)
**Obstetrics and gynecology**			13(2.4)
	Feto**-**maternal medicine		6 (1.1)
	Antenatal care		2 (0.4)
	Female pelvic medicine and reconstructive surgery		2 (0.4)
**Physical medicine and rehabilitation**			11(2.0)
	Physical therapy		4 (0.7)
	Musculoskeletal pain		1 (0.2)
**Ophthalmology**			10 (1.8)
	Glaucoma		2 (0.4)
	Oculoplastic conditions		1 (0.2)
Urology			10 (1.8)
**Infectious disease**			7 (1.3)
	HIV		6 (1.1)
	Tuberculosis		1 (0.2)
Hospice and palliative medicine			6 (1.1)
**Diagnostic radiology**			4 (0.7)
	Ultrasound		1 (0.2)
Anesthesiology			2 (0.4)
**Anatomical and clinical pathology**			
	Digitalization of diagnostic services		1 (0.2)
**Allergies and immunology**			2 (0.4)
	Allergies		2 (0.4)

^a^Number of articles in each medical specialty; where not mentioned, the article is included under the general practice.

^b^Number of articles in each subspecialty; where not mentioned, the article is included in the specialty category only.

^c^Number of articles discussing each specific disease or condition; where not mentioned, the article is included in subspecialty category only.

^d^ECMO: extracorporeal membranous oxygenation.

^e^Articles discussed various aspects of medicine without reference to any specific medical specialty.

In the articles included in our review, the top 5 diseases or conditions for which telehealth was used were diabetes mellitus (9/543, 1.7%), head and neck cancers (8/543, 1.5%), HIV (6/543, 1.1%), epilepsy (5/543, 0.9%), and inflammatory bowel disease (5/543, 0.9%). Within the preventive medicine specialty, 7.7% (42/543) of the articles discussed the various forms of COVID-19 prevention, treatment, and control.

### Classification of Sentiments on Telehealth Use

As described in the Methods, we categorized the sentiments expressed by the authors based on the framework proposed by Nettleton et al [20] and subsequently used by Dol et al [21]. The majority (461/543, 84.9%) of articles were celebratory in nature, followed by those that were contingent (74/543, 13.6%), and concerned (8/543, 1.5%). Articles that were categorized as contingent and concerned predominantly stated the need for more evidence on (1) patient satisfaction, (2) cost**-**effectiveness, (3) efficacy and accuracy of care, and (4) health equity.

## Discussion

### Principal Findings

The findings from our scoping review indicate that substantial published literature on telehealth has emerged and continues to do during the ongoing COVID-19 pandemic. The eagerness of health care providers and expert researchers to share their opinions and research findings on the application and future potential of telehealth is evident. We observed that telehealth remains a topic of interest for a wide variety of journals (generic and specialized). This is not surprising because telehealth not only shows promise but also has the potential to improve health care access globally [25].

The vast majority of published articles in the literature are in the English language. This is also true for articles featuring technology**-**related information, as often there are no suitable words in native languages to define technological advancements. This gap in communication can consequently be a deterrent to publishing, with the views of experts and researchers from non–English-speaking countries being discounted or overlooked [26]. It is interesting to note that most of the articles included in our review had a direct reference to COVID-19 and telehealth in their titles, emphasizing the value and use of telehealth during the pandemic. Although journals usually prefer to publish empirical research and reviews, the fact that the vast majority of the articles in our review were viewpoints and opinions demonstrates the willingness of journals to publish such articles while empirical research on the topic continues to emerge during the pandemic. These experiences, arguments, and debates can help identify future research questions. Although the majority of shorter communications such as opinions, commentaries, and viewpoints may be useful for future research, studies have found that these article types are often not backed by adequate data and/or are poorly reviewed in a rush to disseminate relevant scientific knowledge [27]. The evolving nature of the COVID-19 pandemic necessitates swift publishing; however, the measures to protect scientific integrity cannot be overemphasized [28]. Any follow-up systematic reviews on telehealth during the pandemic should place high emphasis on the quality of studies included. Moreover, the small proportion of empirical research and reviews on telehealth during this period should also be seen as a call for additional scientifically robust primary studies with hard data and statistics to offer current and reliable evidence on telehealth.

Our analysis of the first authors’ affiliations and the study’s geographic focus showed that a vast majority of the publications originated from high-income and upper-middle–income countries in the Americas, Europe, and the Western Pacific regions. The higher number of publications from the United States of America is commensurate with the recent rapid surge in telehealth use seen in that country. This can be attributed to the flexibility provided by the Health Insurance Portability and Accountability Act of 1996 and the willingness of insurance companies to reimburse for the services provided via telehealth [29]. Among the low- and upper-middle–income countries, the geographic focus was on India, China, Brazil, and Iran. These countries are large, have been profoundly affected by the COVID-19 pandemic, and had some telehealth-related infrastructure in place even before the onset of the pandemic. We found that the list of countries with the maximum number of publications, based on our review, was mostly consistent with the country-wise publication output as published by the National Science Board of the United States of America [30]. Interestingly, we found no publication originating from Africa; this may be because COVID-19 has had a relatively low overall impact on health within the continent to date [31] and the fact that telehealth infrastructure availability is limited in many African countries [32].

The wide range and variation in the definitions of telehealth used by the authors, despite WHO’s efforts to standardize the definition, is concerning. This is reflective of the lack of consensus in the scientific world on what constitutes telehealth [8,33]. Given more recent developments in the field, including the growing scope of wearable technology and AI, that can augment telehealth and compensate for some of its limitations (eg, physical examination and continuous monitoring) [34], there is a need to revisit the definition of telehealth and arrive at a global consensus. We believe that the use of very broad terminologies (such as ehealth) or very narrow terminologies (such as telestroke) might hamper the standardization and introduction of legal and regulatory provisions to facilitate the use of telehealth. Uniformity in terminology is also important for future evidence-generating systematic reviews, such as those evaluating the efficiency and effectiveness of telehealth.

### Telehealth and COVID-19

Our study demonstrates that telehealth has been used broadly in the context of the COVID-19 pandemic as an aid to the active management of patients with COVID-19; for surveillance, triage, and diagnosis; treatment including e-prescriptions; follow-up care; and rehabilitation. It is interesting to note that telehealth has been complemented by the use of wearable devices and selfcare equipment, such as glucometers, handheld blood pressure monitors, pulse oximeters, and digital stethoscopes [35,36]. The use of such equipment, as necessitated by the pandemic, has favorably augmented telehealth use. This can be expected to continue to serve as an adjunct to the provision of telehealth and in-person health care delivery in the long-term [37]. Futuristic advancements in the development and deployment of wearable devices and unobtrusive sensing systems in telehealth offer considerable scope for potential applications and research [35].

Our findings suggest that telehealth has been extensively utilized for medical education during the COVID-19 pandemic. Its use must have considerably helped medical schools in the delivery and continuity of medical education and training. It must also have allowed students to keep on track so that they are able to complete graduation requirements in a timely manner despite challenges associated with the pandemic. Telehealth has been previously used for case rounds and case discussions in residency training [38]. Case discussions, in particular, involve an intersection between tele-education and telehealth, as they bring expert (clinical) educators closer to trainees and students based in remote locations [39]. Certain medical specialties such as dermatology, neurology, obstetrics and gynecology, orthopedics, pathology, psychiatry, and surgery were more likely to use telehealth in residency training than other specialties [40]. Moreover, almost all medical specialties have tapped into telehealth for medical care. Specialties such as dermatology, pathology, and psychiatry have long employed telehealth to provide services to patients [7], whereas other specialties such as surgery, anesthesiology, and oncology are beginning to find telehealth useful at least to deliver certain components of their regular service.

Our findings suggest telehealth has been used to manage a wide spectrum of noncommunicable and communicable diseases, including COVID-19. An earlier scoping review had identified the predominant use of telehealth for noncommunicable diseases, but it did not focus on communicable diseases [41]. In addition to supporting disease-specific management, telehealth has also been extended to provide holistic medical care to specific target groups such as adolescents and older people [42,43].

Given the large scope of telehealth services analyzed by our study, it was no surprise to find that authors of an overwhelming majority of published articles expressed a celebratory sentiment regarding the use of telehealth. This finding is in line with that of many past reviews on the subject [9,44-46]. Authors of the few articles that expressed contingent and concerned sentiments cited the need to review additional evidence on the use of telehealth for health care delivery and to further explore ethics and equity in the context of telehealth use [47-51]. Given that the majority of articles were opinions and perspectives, this is a concern that cannot be overlooked.

### Study Limitations

Although this scoping review was conducted according to the suggested methodology, we acknowledge our study has some limitations. We searched only 2 databases and did not actively search the grey literature and preprints. Consequently, our search may not have been exhaustive. Furthermore, following best practices in scoping reviews would have required us to have 2 independent reviewers involved in the screening and data extraction or charting stages. Given the time sensitivity and high volume of publications, an optimized approach had to be considered. However, we have ensured transparency by clearly outlining the process followed in the Methods section. Although the initial title and abstract screening was performed by 1 reviewer, given the large number of studies for data charting, the full-text screening for data extraction was completed by 2 reviewers. Any variation between the 2 reviewers would have been mitigated by the standardized, well-defined, self-explanatory data extraction form used. As most of the data (except for the sentiment analysis) extracted from the articles contained factual information (such as the name of the journal, type of publication, country of affiliation of first author) drawn directly from the articles and were objectively verifiable, the likelihood of variation remained low. The fact that both the reviewers cross-verified at least 10% of each other’s work provides an added level of cushion to the process. Another limitation to this scoping review is the rapidity with which articles seem to be published. From the time of our search to the writing of the manuscript, the number of publications in PubMed alone had almost doubled. As a result, this review is expected to serve the purpose of being an interim scoping review only and can be further updated as the need arises.

### Conclusions

Our scoping review highlights the exponential use of telehealth during a defined period of the ongoing COVID-19 pandemic. Experiential reports pertaining to telehealth use are being published extensively, albeit mostly from high-income countries such as the United States, in particular. A wide variety of journals, including specialty journals, are increasingly publishing more material on telehealth. This is in tandem with an increasing number of medical specialties beginning to use telehealth for patient care. Our study also found many subspecialties of medicine that utilize telehealth. Emerging technologies, including wearable devices and AI, are futuristic adjuncts to telehealth, which may help mitigate some of its limitations. The positive sentiment expressed by most authors regarding the use of telehealth is reflective of the developing enthusiasm and receptiveness for this technology. However, we cannot overlook the need for additional robust evidence on the safety and effectiveness of telehealth as compared to the traditional health care delivery model, as pointed out by some authors. Our scoping review demonstrates the breadth and depth of data currently being generated in this area and will enable future systematic reviews and meta-analyses to help address research gaps and answer emerging questions.

Telehealth may have a significant effect in advancing health care in the future. If it has the potential to transform health care, we must ensure that low- and middle-income countries can benefit from it. More North-South and South-South collaborations between academics and practitioners are needed to establish the feasibility and utility of telehealth in resource-limited settings. The advances in medical education facilitated through telehealth are noteworthy. The school curricula for health professionals must be reformed to include specific education pertaining to health care delivery utilizing telehealth, as this can have a profound effect on patient outcomes and the overall health of the population. Continuing medical education and continuing professional development in telehealth use must also concurrently be offered to practicing health care practitioners, so they can become cognizant of and comfortable with using this modality to aid the provision of health care delivery. Given the rapidity with which telehealth is advancing, a global consensus on definitions, boundaries, protocols, monitoring, evaluation, and data privacy are urgently needed.

## Appendix

Multimedia Appendix 1 Preferred Reporting Items for Systematic reviews and Meta-Analyses extension for Scoping Reviews (PRISMA-ScR) checklist.

Multimedia Appendix 2 Search strategy utilized in the scoping review.

Multimedia Appendix 3 List of published articles included in the scoping review.

Multimedia Appendix 4 Classification of the country of affiliation of the first author and the country of focus of the study.

Multimedia Appendix 5 Various definitions of telehealth and its variants used in the studies included in the scoping review.

## Abbreviations

AI: artificial intelligence
ICT: Information and Communication Technology
mhealth: mobile health
PRISMA-ScR: Preferred Reporting Items for Systematic reviews and Meta-Analyses Extension for Scoping Reviews
WHO: World Health Organization
