# Supplementary material for: Use of Telehealth During the COVID-19 Pandemic: Scoping Review
Source: J Med Internet Res. 2020 Dec 1;22(12):e24087. doi: 10.2196/24087 (PMC7710390; doi:10.2196/24087)
Supplement: Multimedia Appendix 2 [file jmir_v22i12e24087_app2.docx]

**Multimedia appendix 2**

| **Search number** | **Query** | **Results** | **Date** |
| --- | --- | --- | --- |
| 1 | (telemedicine[MeSH Terms]) OR (telehealth[MeSH Terms]) | 28,154 | Wednesday, June 10, 2020 |
| 2 | mobile health[MeSH Terms] | 28,154 | Wednesday, June 10, 2020 |
| 3 | "tele*"[All Fields] OR "digital*"[All Fields] OR "remote*"[All Fields] OR "video*"[All Fields] OR "Ehealth"[All Fields] OR "e-health"[All Fields] OR "e-health"[All Fields] OR "electronic health"[All Fields] OR "virtual"[All Fields] OR "virtuality"[All Fields] OR "virtualization"[All Fields] OR "virtualized"[All Fields] OR "virtualizing"[All Fields] OR "virtuals"[All Fields] | 697,903 | Wednesday, June 10, 2020 |
| 4 | #1 OR #2 OR #3 | 697,903 | Wednesday, June 10, 2020 |
| 5 | (((((("covid 19"[All Fields] OR "covid 2019"[All Fields]) OR "severe acute respiratory syndrome coronavirus 2"[Supplementary Concept]) OR "severe acute respiratory syndrome coronavirus 2"[All Fields]) OR "2019 ncov"[All Fields]) OR "sars cov 2"[All Fields]) OR "2019ncov"[All Fields]) OR (("wuhan"[All Fields] AND ("coronavirus"[MeSH Terms] OR "coronavirus"[All Fields])) AND (2019/12/1:2019/12/31[Date - Publication] OR 2020/1/1:2020/12/31[Date - Publication])) | 21,172 | Wednesday, June 10, 2020 |
| 6 | #4 AND #5 | **1,362** | Wednesday, June 10, 2020 |
|  |  |  |  |

*PubMed - Wednesday, June 10, 2020*

|  | Note: Search strategy was modified from: Ming WK, Mackillop LH, Farmer AJ, et al. Telemedicine Technologies for Diabetes in Pregnancy: A Systematic Review and Meta Analysis. J Med Internet Res. 2016;18(11):e290. Published 2016 Nov 9. doi:10.2196/jmir.6556 |
| --- | --- |

| **Search number** | **Query** | **Comments** | **Results** | **Date** |
| --- | --- | --- | --- | --- |
| 1 | exp telehealth/ |  | 44,715 | Wednesday, June 10, 2020 |
| 2 | exp telemedicine/ |  | 39,145 | Wednesday, June 10, 2020 |
| 3 | (tele* or digital* or remote or video* or Ehealth or e-health or "electronic health" or virtual).mp. [mp=title, abstract, heading word, drug trade name, original title, device manufacturer, drug manufacturer, device trade name, keyword, floating subheading word, candidate term word] |  | 803,431 | Wednesday, June 10, 2020 |
| 4 | 1 or 2 or 3 |  | 803,431 | Wednesday, June 10, 2020 |
| 5 | limit 4 to exclude medline journals |  | 75,514 | Wednesday, June 10, 2020 |
| 6 | limit 5 to covid-19 |  | **118** | Wednesday, June 10, 2020 |

*Embase - Wednesday, June 10, 2020*
