# Supplementary material for: Use of Telehealth During the COVID-19 Pandemic: Scoping Review
Source: J Med Internet Res. 2020 Dec 1;22(12):e24087. doi: 10.2196/24087 (PMC7710390; doi:10.2196/24087)
Supplement: Multimedia Appendix 3 [file jmir_v22i12e24087_app3.pdf]

Multimedia Appendix 3

1. Aafjes-van Doorn K, Bekes V, Prout TA, Hoffman L. Psychotherapists' vicarious traumatization during the COVID-19 pandemic. Psychol Trauma. 2020;12(S1):S148-S50.

2. Abraham WT, Fiuzat M, Psotka MA, O'Connor CM. Heart Failure Collaboratory Statement on Clinical Trials in the Landscape of COVID-19. JACC Heart Fail. 2020;8(5):423-5.

3. Adams C. Goals of Care in a Pandemic: Our Experience and Recommendations. J Pain Symptom Manage. 2020;60(1):e15-e7.

4. Adan GH, Mitchell JW, Marson T. Epilepsy care in the COVID-19 era. Clin Med (Lond). 2020;20(4):e104-e6.

5. Agarwal S, Sabadia S, Abou-Fayssal N, Kurzweil A, Balcer LJ, Galetta SL. Training in neurology: Flexibility and adaptability of a neurology training program at the epicenter of COVID-19. Neurology. 2020;94(24):e2608-e14.

6. Aghakhani K, Shalbafan M. What COVID-19 outbreak in Iran teaches us about virtual medical education. Med Educ Online. 2020;25(1):1770567.

7. Ahmadi A, Ghafour I, Hosseini SH, Hashemvarzi M. Psychological interventions for covid-19 outbreak in mazandaran province, iran. Archives of Clinical Infectious Diseases. 2020;15(2):e103415.

8. Ahmady S, Shahbazi S, Heidari M. Transition to Virtual Learning During the Coronavirus Disease-2019 Crisis in Iran: Opportunity Or Challenge? Disaster Med Public Health Prep. 2020:1-2.

9. Ahn DT. The COVID-19 Pandemic: A "Tech"-tonic Shift Toward Virtual Diabetes Care. J Diabetes Sci Technol. 2020;14(4):708-9.

10. Akkara JD, Kuriakose A. Commentary: Gamifying teleconsultation during COVID-19 lockdown. Indian J Ophthalmol. 2020;68(6):1013-4.

11. Al Hussona M, Maher M, Chan D, Micieli JA, Jain JD, Khosravani H, et al. The Virtual Neurologic Exam: Instructional Videos and Guidance for the COVID-19 Era. Can J Neurol Sci. 2020;47(5):598-603.

12. Al Kasab S, Almallouhi E, Holmstedt CA. Optimizing the Use Of Teleneurology During the COVID-19 Pandemic. Telemed J E Health. 2020;26(10):1197-8.

13. Al-Shamsi HO, Alhazzani W, Alhuraiji A, Coomes EA, Chemaly RF, Almuhanna M, et al. A Practical Approach to the Management of Cancer Patients During the Novel Coronavirus Disease 2019 (COVID-19) Pandemic: An International Collaborative Group. Oncologist. 2020;25(6):e936-e45.

14. Alexander M. Let's conquer COVID-19 and sustain our abilities. Spinal Cord Ser Cases. 2020;6(1):19.

15. Ali FR, Al-Niaimi F. Noncutaneous considerations of COVID-19 for dermatology practices. J Cosmet Dermatol. 2020;19(7):1544.

16. Allocca M, Fiorino G, Furfaro F, Gilardi D, Radice S, D'Amico F, et al. Maintaining the Quality Standards of Care for Inflammatory Bowel Disease Patients During the COVID-19 Pandemic. Clin Gastroenterol Hepatol. 2020;18(8):1882-3.

17. Almarzooq ZI, Lopes M, Kochar A. Virtual Learning During the COVID-19 Pandemic: A Disruptive Technology in Graduate Medical Education. J Am Coll Cardiol. 2020;75(20):2635-8.

18. Alsharaydeh I, Rawashdeh H, Saadeh N, Obeidat B, Obeidat N. Challenges and solutions for maternity and gynecology services during the COVID-19 crisis in Jordan. Int J Gynaecol Obstet. 2020;150(2):159-62.

19. Alvarez-Roman MT, De la Corte-Rodriguez H, Rodriguez-Merchan EC, Martin-Salces M, Rivas-Pollmar MI, Butta NV, et al. COVID-19 and telemedicine in hemophilia in a patient with severe hemophilia A and orthopedic surgery. Haemophilia. 2020.

20. Alwashmi MF. The Use of Digital Health in the Detection and Management of COVID-19. Int J Environ Res Public Health. 2020;17(8).

21. Amatya S, Corr TE, Gandhi CK, Glass KM, Kresch MJ, Mujsce DJ, et al. Management of newborns exposed to mothers with confirmed or suspected COVID-19. J Perinatol. 2020;40(7):987-96.

22. American association for cancer research. Cancer Care Goes Virtual in Response to COVID-19. Cancer Discov. 2020;10(6):755.

23. An TW, Henry JK, Igboechi O, Wang P, Yerrapragada A, Lin CA, et al. How Are Orthopaedic Surgery Residencies Responding to the COVID-19 Pandemic? An Assessment of Resident Experiences in Cities of Major Virus Outbreak. J Am Acad Orthop Surg.

24. Andrews JA, Berry JD, Baloh RH, Carberry N, Cudkowicz ME, Dedi B, et al. Amyotrophic lateral sclerosis care and research in the United States during the COVID-19 pandemic: Challenges and opportunities. Muscle Nerve. 2020;62(2):182-6.

25. Andrikopoulos S, Johnson G. The Australian response to the COVID-19 pandemic and diabetes - Lessons learned. Diabetes Res Clin Pract. 2020;165:108246.

26. Annis T, Pleasants S, Hultman G, Lindemann E, Thompson JA, Billecke S, et al. Rapid implementation of a COVID-19 remote patient monitoring program. J Am Med Inform Assoc. 2020;27(8):1326-30.

27. Anwar A, Seger C, Tollefson A, Diachun CAB, Tanaka P, Umar S. Medical education in the COVID-19 era: Impact on anesthesiology trainees. J Clin Anesth. 2020;66:109949.

28. Appireddy R, Jalini S, Shukla G, Boisse Lomax L. Tackling the Burden of Neurological Diseases in Canada with Virtual Care During the COVID-19 Pandemic and Beyond. Can J Neurol Sci. 2020;47(5):594-7.

29. Aranda-Narvaez JM, Tallon-Aguilar L, Pareja-Ciuro F, Martin-Martin G, Gonzalez-Sanchez AJ, Rey-Simo I, et al. [Emergency Surgery and Trauma Care During COVID-19 Pandemic. Recommendations of the Spanish Association of Surgeons]. Cir Esp. 2020;98(8):433-41.

30. Arends MJ, Salto-Tellez M. Low-contact and high-interconnectivity pathology (LC&HI Path): post-COVID19-pandemic practice of pathology. Histopathology. 2020;77(4):518-24.

31. Argento NB. A Pandemic Forces United States to Leap Forward. J Diabetes Sci Technol. 2020;14(4):710-1.

32. Armitage L, Lawson BK, Whelan ME, Newhouse N. Paying SPECIAL consideration to the digital sharing of information during the COVID-19 pandemic and beyond. BJGP Open. 2020;4(2).

33. Arnaez J, Montes MT, Herranz-Rubia N, Garcia-Alix A. The Impact of the Current SARS-CoV-2 Pandemic on Neonatal Care. Front Pediatr. 2020;8:247.

34. Arneson SL, Tucker SJ, Mercier M, Singh J. Answering the Call: Impact of Tele-ICU Nurses During the COVID-19 Pandemic. Crit Care Nurse. 2020;40(4):25-31.

35. Arnold RH, Tideman PA, Devlin GP, Carroll GE, Elder A, Lowe H, et al. Rural and Remote Cardiology During the COVID-19 Pandemic: Cardiac Society of Australia and New Zealand (CSANZ) Consensus Statement. Heart Lung Circ. 2020;29(7):e88-e93.

36. Arrese M. Telemedicine, COVID-19 and liver diseases: Revamping remote care initiatives in hepatology. Ann Hepatol. 2020;19(4):339-40.

37. Arsand E. The COVID-19 Pandemic Revealed the Importance and Shortcomings of Technologies for Diabetes Support. J Diabetes Sci Technol. 2020;14(4):712-3.

38. Ashokka B, Ong SY, Tay KH, Loh NHW, Gee CF, Samarasekera DD. Coordinated responses of academic medical centres to pandemics: Sustaining medical education during COVID-19. Med Teach. 2020;42(7):762-71.

39. Aslani N, Garav, A. The role of telemedicine to control COVID-19. Archives of Clinical Infectious Diseases. 2020;15:e102949.

40. Aung MN, Yuasa M, Koyanagi Y, Aung TNN, Moolphate S, Matsumoto H, et al. Sustainable health promotion for the seniors during COVID-19 outbreak: a lesson from Tokyo. J Infect Dev Ctries. 2020;14(4):328-31.

41. Aziz A, Zork N, Aubey JJ, Baptiste CD, D'Alton ME, Emeruwa UN, et al. Telehealth for High-Risk Pregnancies in the Setting of the COVID-19 Pandemic. Am J Perinatol. 2020;37(8):800-8.

42. Azizy A, Fayaz M, Agirbasli M. Do Not Forget Afghanistan in Times of COVID-19: Telemedicine and the Internet of Things to Strengthen Planetary Health Systems. OMICS. 2020;24(6):311-3.

43. Bacq S, Geoghegan W, Josefy M, Stevenson R, Williams TA. The COVID-19 Virtual Idea Blitz: Marshaling social entrepreneurship to rapidly respond to urgent grand challenges. Bus Horiz. 2020.

44. Bae YS, Kim KH, Choi SW, Ko T, Jeong CW, Cho B, et al. Information Technology-Based Management of Clinically Healthy COVID-19 Patients: Lessons From a Living and Treatment Support Center Operated by Seoul National University Hospital. J Med Internet Res. 2020;22(6):e19938.

45. Bamias G, Lagou S, Gizis M, Karampekos G, Kyriakoulis KG, Pontas C, et al. The Greek Response to COVID-19: A True Success Story from an IBD Perspective. Inflamm Bowel Dis. 2020;26(8):1144-8.

46. Banerjee M, Chakraborty S, Pal R. Teleconsultation and Diabetes Care Amid COVID-19 Pandemic in India: Scopes and Challenges. J Diabetes Sci Technol. 2020;14(4):714-5.

47. Barber TM. COVID-19 and diabetes mellitus: implications for prognosis and clinical management. Expert Rev Endocrinol Metab. 2020;15(4):227-36.

48. Barney A, Buckelew S, Mesheriakova V, Raymond-Flesch M. The COVID-19 Pandemic and Rapid Implementation of Adolescent and Young Adult Telemedicine: Challenges and Opportunities for Innovation. J Adolesc Health. 2020;67(2):164-71.

49. Barsom EZ, Feenstra TM, Bemelman WA, Bonjer JH, Schijven MP. Coping with COVID-19: scaling up virtual care to standard practice. Nat Med. 2020;26(5):632-4.

50. Bartels SJ, Baggett TP, Freudenreich O, Bird BL. COVID-19 Emergency Reforms in Massachusetts to Support Behavioral Health Care and Reduce Mortality of People With Serious Mental Illness. Psychiatr Serv. 2020;71(10):1078-81.

51. Basch CH, Hillyer GC, Meleo-Erwin ZC, Jaime C, Mohlman J, Basch CE. Preventive Behaviors Conveyed on YouTube to Mitigate Transmission of COVID-19: Cross-Sectional Study. JMIR Public Health Surveill. 2020;6(2):e18807.

52. Bashshur R, Doarn CR, Frenk JM, Kvedar JC, Woolliscroft JO. Telemedicine and the COVID-19 Pandemic, Lessons for the Future. Telemed J E Health. 2020;26(5):571-3.

53. Basil GW, Eichberg DG, Perez-Dickens M, Menendez I, Ivan ME, Urakov T, et al. Letter: Implementation of a Neurosurgery Telehealth Program Amid the COVID-19 Crisis-Challenges, Lessons Learned, and a Way Forward. Neurosurgery. 2020;87(2):E260-E2.

54. Bassi A, Arfin S, John O, Jha V. An overview of mobile applications (apps) to support the coronavirus disease 2019 response in India. Indian J Med Res. 2020;151(5):468-73.

55. Bastier PL, Aisenberg N, Durand F, Lestang P, Abedipour D, Gallet de Santerre O, et al. Treatment of sleep apnea by ENT specialists during the COVID-19 pandemic. Eur Ann Otorhinolaryngol Head Neck Dis. 2020;137(4):319-21.

56. Basu S. Non-communicable disease management in vulnerable patients during Covid-19. Indian J Med Ethics. 2020;V(2):103-5.

57. Batra PS, LoSavio PS, Michaelides E, Revenaugh PC, Tajudeen BA, Al-Khudari S, et al. Management of the Clinical and Academic Mission in an Urban Otolaryngology Department During the COVID-19 Global Crisis. Otolaryngol Head Neck Surg. 2020;163(1):162-9.

58. Bayram M, Springer S, Garvey CK, Ozdemir V. COVID-19 Digital Health Innovation Policy: A Portal to Alternative Futures in the Making. OMICS. 2020;24(8):460-9.

59. Beaman A, Davidson PM. Global service-learning and COVID-19-What the future might look like? J Clin Nurs. 2020.

60. Beaunoyer E, Dupere S, Guitton MJ. COVID-19 and digital inequalities: Reciprocal impacts and mitigation strategies. Comput Human Behav. 2020;111:106424.

61. Behbahani S, Smith CA, Carvalho M, Warren CJ, Gregory M, Silva NA. Vulnerable Immigrant Populations in the New York Metropolitan Area and COVID-19: Lessons Learned in the Epicenter of the Crisis. Acad Med. 2020.

62. Berg EA, Picoraro JA, Miller SD, Srinath A, Franciosi JP, Hayes CE, et al. COVID-19-A Guide to Rapid Implementation of Telehealth Services: A Playbook for the Pediatric Gastroenterologist. J Pediatr Gastroenterol Nutr. 2020;70(6):734-40.

63. Bergman D, Bethell C, Gombojav N, Hassink S, Stange KC. Physical Distancing With Social Connectedness. Ann Fam Med. 2020;18(3):272-7.

64. Bilal, Latif F, Bashir MF, Komal B, Tan D. Role of electronic media in mitigating the psychological impacts of novel coronavirus (COVID-19). Psychiatry Res. 2020;289:113041.

65. Bilato C, Roncon L, Anselmi M, Valle R, Perrone C, Mecenero A, et al. [Managing cardiac patients post-COVID-19 pandemic: a proposal by the ANMCO Veneto Region]. G Ital Cardiol (Rome). 2020;21(6):408-16.

66. Bini SA, Schilling PL, Patel SP, Kalore NV, Ast MP, Maratt JD, et al. Digital Orthopaedics: A Glimpse Into the Future in the Midst of a Pandemic. J Arthroplasty. 2020;35(7S):S68-S73.

67. Birkeland KI. Some Lessons Learned About Diabetes and COVID-19 During the Early Stage of the Epidemic in Norway. J Diabetes Sci Technol. 2020;14(4):718-9.

68. Blake H, Bermingham F, Johnson G, Tabner A. Mitigating the Psychological Impact of COVID-19 on Healthcare Workers: A Digital Learning Package. Int J Environ Res Public Health. 2020;17(9).

69. Blankenburg R, Poitevien P, Gonzalez Del Rey J, Degnon L, Virtual Cafe Study T. Virtual Cafes: An Innovative Way for Rapidly Disseminating Educational Best Practices and Building Community During COVID-19. Acad Pediatr. 2020;20(6):756-7.

70. Blue R, Yang AI, Zhou C, De Ravin E, Teng CW, Arguelles GR, et al. Telemedicine in the Era of Coronavirus Disease 2019 (COVID-19): A Neurosurgical Perspective. World Neurosurg. 2020;139:549-57.

71. Boccalatte LA, Larranaga JJ, Perez Raffo GM, Teijido CA, Garcia Fornari G, Staneloni MI, et al. Brief guideline for the prevention of COVID-19 infection in head and neck and otolaryngology surgeons. Am J Otolaryngol. 2020;41(3):102484.

72. Bocher R, Jansen C, Gayet P, Gorwood P, Laprevote V. [Responsiveness and sustainability of psychiatric care in France during COVID-19 epidemic]. Encephale. 2020;46(3S):S81-S4.

73. Boehm K, Ziewers S, Brandt MP, Sparwasser P, Haack M, Willems F, et al. Telemedicine Online Visits in Urology During the COVID-19 Pandemic-Potential, Risk Factors, and Patients' Perspective. Eur Urol. 2020;78(1):16-20.

74. Boelaert K, Visser WE, Taylor PN, Moran C, Leger J, Persani L. ENDOCRINOLOGY IN THE TIME OF COVID-19: Management of hyperthyroidism and hypothyroidism. Eur J Endocrinol. 2020;183(1):G33-G9.

75. Boettler T, Newsome PN, Mondelli MU, Maticic M, Cordero E, Cornberg M, et al. Care of patients with liver disease during the COVID-19 pandemic: EASL-ESCMID position paper. JHEP Rep. 2020;2(3):100113.

76. Bonora BM, Boscari F, Avogaro A, Bruttomesso D, Fadini GP. Glycaemic Control Among People with Type 1 Diabetes During Lockdown for the SARS-CoV-2 Outbreak in Italy. Diabetes Therapy. 2020.

77. Borchert A, Baumgarten L, Dalela D, Jamil M, Budzyn J, Kovacevic N, et al. Managing Urology Consultations During COVID-19 Pandemic: Application of a Structured Care Pathway. Urology. 2020;141:7-11.

78. Boukhris M, Hillani A, Moroni F, Annabi MS, Addad F, Ribeiro MH, et al. Cardiovascular Implications of the COVID-19 Pandemic: A Global Perspective. Can J Cardiol. 2020;36(7):1068-80.

79. Boulton C. Supporting People With Diabetes, Through COVID-19 and Beyond. J Diabetes Sci Technol. 2020;14(4):720.

80. Bowe T, Hunter DG, Mantagos IS, Kazlas M, Jastrzembski BG, Gaier ED, et al. Virtual Visits in Ophthalmology: Timely Advice for Implementation During the COVID-19 Public Health Crisis. Telemed J E Health. 2020;26(9):1113-7.

81. Bozzalla Cassione E, Zanframundo G, Biglia A, Codullo V, Montecucco C, Cavagna L. COVID-19 infection in a northern-Italian cohort of systemic lupus erythematosus assessed by telemedicine. Ann Rheum Dis. 2020;79(10):1382-3.

82. Brawner BM. #SendHelpNow: Mental wellness and virtual connection in the age of coronavirus. J Psychiatr Ment Health Nurs. 2020.

83. Bressler MY, Siegel DM, Markowitz O. Virtual dermatology: a COVID-19 update. Cutis. 2020;105(4):163-4;E2.

84. Brigo F, Bonavita S, Leocani L, Tedeschi G, Lavorgna L. Telemedicine and the challenge of epilepsy management at the time of COVID-19 pandemic. Epilepsy Behav.110:107164.

85. Brough HA, Kalayci O, Sediva A, Untersmayr E, Munblit D, Rodriguez Del Rio P, et al. Managing childhood allergies and immunodeficiencies during respiratory virus epidemics - The 2020 COVID-19 pandemic: A statement from the EAACI-section on pediatrics. Pediatr Allergy Immunol. 2020.

86. Brunasso AMG, Massone C. Teledermatologic monitoring for chronic cutaneous autoimmune diseases with smartworking during COVID-19 emergency in a tertiary center in Italy. Dermatol Ther. 2020:e13495.

87. Brunetti O, Derakhshani A, Baradaran B, Galvano A, Russo A, Silvestris N. COVID-19 Infection in Cancer Patients: How Can Oncologists Deal With These Patients? Frontiers in Oncology. 2020;10:734.

88. Brunetti-Pierri N, Fecarotta S, Staiano A, Strisciuglio P, Parenti G. Ensuring continuity of care for children with inherited metabolic diseases at the time of COVID-19: the experience of a metabolic unit in Italy. Genet Med. 2020;22(7):1178-80.

89. Bryant MS, Fedson SE, Sharafkhaneh A. Using Telehealth Cardiopulmonary Rehabilitation during the COVID-19 Pandemic. J Med Syst. 2020;44(7):125.

90. Buckley H. Faculty development in the COVID-19 pandemic: So close - yet so far. Med Educ. 2020.

91. Butel P. Telehealth: Synopsis. Aust J Gen Pract. 2020;49.

92. Caetano R, Silva AB, Guedes A, Paiva CCN, Ribeiro GDR, Santos DL, et al. Challenges and opportunities for telehealth during the COVID-19 pandemic: ideas on spaces and initiatives in the Brazilian context. Cad Saude Publica. 2020;36(5):e00088920.

93. Cai T, Verze P, Luciani L, Malossini G, Bjerklund Johansen TE, Benetollo PP, et al. What do patients say about telephone-based urological consultations at the time of the COVID-19 pandemic? Minerva Urol Nefrol. 2020;72(4):515-6.

94. Calton B, Abedini N, Fratkin M. Telemedicine in the Time of Coronavirus. J Pain Symptom Manage. 2020;60(1):e12-e4.

95. Capra R, Mattioli F. Tele-health in neurology: an indispensable tool in the management of the SARS-CoV-2 epidemic. J Neurol. 2020;267(7):1885-6.

96. Carlson JL, Goldstein R. Using the Electronic Health Record to Conduct Adolescent Telehealth Visits in the Time of COVID-19. J Adolesc Health. 2020;67(2):157-8.

97. Carr D, Boerner K, Moorman S. Bereavement in the Time of Coronavirus: Unprecedented Challenges Demand Novel Interventions. J Aging Soc Policy. 2020;32(4-5):425-31.

98. Carr E. eHealth During a Pandemic. Clin J Oncol Nurs. 2020;24(3):3.

99. Cartron AM, Rismiller K, Trinidad JCL. Store-and-forward teledermatology in the era of COVID-19: A pilot study. Dermatol Ther. 2020:e13689.

100. Casas RS, Cooper JL, Hempel EV. COVID-19 risk triage: Engaging residents in telephonic screening. Med Educ. 2020;54(7):670.

101. Casella G, Ingravalle F, Ingravalle A, Monti C, Bonetti F, Limonta A. COVID emergency: an opportunity to increase the interaction between hepatologist and primary care physician. Minerva Gastroenterol Dietol. 2020.

102. Celesti A, Ruggeri A, Fazio M, Galletta A, Villari M, Romano A. Blockchain-Based Healthcare Workflow for Tele-Medical Laboratory in Federated Hospital IoT Clouds. Sensors (Basel). 2020;20(9).

103. Ceravolo MG, de Sire A, Andrenelli E, Negrini F, Negrini S. Systematic rapid "living" review on rehabilitation needs due to COVID-19: update to March 31st, 2020. Eur J Phys Rehabil Med. 2020;56(3):347-53.

104. Ceriello A, Schnell O. COVID-19: Considerations of Diabetes and Cardiovascular Disease Management. J Diabetes Sci Technol. 2020;14(4):723-4.

105. Cervino G, Oteri G. COVID-19 Pandemic and Telephone Triage before Attending Medical Office: Problem or Opportunity? Medicina (Kaunas). 2020;56(5).

106. Chandra S, Laoteppitaks C, Mingioni N, Papanagnou D. Zooming-out COVID-19: Virtual clinical experiences in an emergency medicine clerkship. Med Educ. 2020.

107. Chang MC, Boudier-Reveret M. Usefulness of Telerehabilitation for Stroke Patients During the COVID-19 Pandemic. Am J Phys Med Rehabil. 2020;99(7):582.

108. Chatterjee SS, Barikar CM, Mukherjee A. Impact of COVID-19 pandemic on pre-existing mental health problems. Asian J Psychiatr. 2020;51:102071.

109. Chauhan V, Galwankar S, Arquilla B, Garg M, Somma SD, El'Menyar A, et al. Novel coronavirus (COVID-19): Leveraging telemedicine to optimize care while minimizing exposures and viral transmission. Journal of Emergencies, Trauma and Shock. 2020;13(1):20-4.

110. Chawla S. COVID-19: Challenges and opportunities for dermatology response. J Dermatolog Treat. 2020;31(4):326.

111. Chenneville T, Schwartz-Mette R. Ethical considerations for psychologists in the time of COVID-19. Am Psychol. 2020;75(5):644-54.

112. Chevance A, Gourion D, Hoertel N, Llorca PM, Thomas P, Bocher R, et al. Ensuring mental health care during the SARS-CoV-2 epidemic in France: A narrative review. Encephale. 2020;46(3):193-201.

113. Chick RC, Clifton GT, Peace KM, Propper BW, Hale DF, Alseidi AA, et al. Using Technology to Maintain the Education of Residents During the COVID-19 Pandemic. J Surg Educ. 2020;77(4):729-32.

114. Chiolero A. Covid-19: a digital epidemic. BMJ. 2020;368:m764.

115. Chou E, Hsieh YL, Wolfshohl J, Green F, Bhakta T. Onsite telemedicine strategy for coronavirus (COVID-19) screening to limit exposure in ED. Emerg Med J. 2020;37(6):335-7.

116. Chowdhury D, Datta D. Managing Migraine in the Times of COVID-19 Pandemic. Ann Indian Acad Neurol. 2020;23(Suppl 1):S33-S9.

117. Christ-Crain M, Hoorn EJ, Sherlock M, Thompson CJ, Wass JAH. ENDOCRINOLOGY IN THE TIME OF COVID-19: Management of diabetes insipidus and hyponatraemia. Eur J Endocrinol. 2020;183(1):G9-G15.

118. Christensen L, Rasmussen CS, Benfield T, Franc JM. A Randomized Trial of Instructor-Led Training Versus Video Lesson in Training Health Care Providers in Proper Donning and Doffing of Personal Protective Equipment. Disaster Med Public Health Prep. 2020:1-15.

119. Chua MLK, Ma DJ, Anderson CM, Karam SD, Margalit DN, Kimple RJ. Follow-Up and Management of Patients With Head and Neck Cancer During the 2019 Novel Coronavirus (SARS-CoV-2) Disease Pandemic. Adv Radiat Oncol. 2020;5(4):631-6.

120. Cinelli E, Fabbrocini G, Fattore D, Marasca C, Damiani G, Annunziata MC. Safe distance, safe patients! Therapeutic management of oncological patients affected by cutaneous and mucosal adverse events during the COVID-19 pandemic: an Italian experience. Support Care Cancer. 2020;28(9):3991-3.

121. Cinelli E, Megna M, Di Guida A, Greco V, Annunziata MC, Fabbrocini G. Teledermatology for patient management, dermatology education and research during the COVID-19 pandemic. Australas J Dermatol. 2020.

122. Cleland JGF, Clark RA, Pellicori P, Inglis SC. Caring for people with heart failure and many other medical problems through and beyond the COVID-19 pandemic: the advantages of universal access to home telemonitoring. Eur J Heart Fail. 2020;22(6):995-8.

123. Codispoti CD, Bandi S, Moy JN, Mahdavinia M. Running a virtual allergy division and training program in the time of COVID-19 pandemic. J Allergy Clin Immunol. 2020;145(5):1357-9.

124. Cohen BH, Busis NA, Ciccarelli L. Coding in the World of COVID-19: Non-Face-to-Face Evaluation and Management Care. Continuum (Minneap Minn). 2020;26(3):785-98.

125. Colle R, Ait Tayeb AEK, de Larminat D, Commery L, Boniface B, Lasica PA, et al. Short-term acceptability by patients and psychiatrists of the turn to psychiatric teleconsultation in the context of the COVID-19 pandemic. Psychiatry Clin Neurosci. 2020;74(8):443-4.

126. Collier S. A Geriatric Psychiatry Virtual Rotation During Covid-19. Am J Geriatr Psychiatry. 2020;28(8):891.

127. Compton M, Soper M, Reilly B, Gettle L, List R, Bailey M, et al. A Feasibility Study of Urgent Implementation of Cystic Fibrosis Multidisciplinary Telemedicine Clinic in the Face of COVID-19 Pandemic: Single-Center Experience. Telemed J E Health. 2020;26(8):978-84.

128. Connor MJ, Winkler M, Miah S. COVID-19 pandemic - is virtual urology clinic the answer to keeping the cancer pathway moving? BJU Int. 2020;125(6):E3-E4.

129. Contreras CM, Metzger GA, Beane JD, Dedhia PH, Ejaz A, Pawlik TM. Telemedicine: Patient-Provider Clinical Engagement During the COVID-19 Pandemic and Beyond. J Gastrointest Surg. 2020;24(7):1692-7.

130. Corden E, Rogers AK, Woo WA, Simmonds R, Mitchell CD. A targeted response to the COVID-19 pandemic: analysing effectiveness of remote consultations for triage and management of routine dermatology referrals. Clin Exp Dermatol. 2020.

131. Cormi C, Chrusciel J, Laplanche D, Drame M, Sanchez S. Telemedicine in nursing homes during the COVID-19 outbreak: A star is born (again). Geriatr Gerontol Int. 2020;20(6):646-7.

132. Corruble E. A Viewpoint From Paris on the COVID-19 Pandemic: A Necessary Turn to Telepsychiatry. J Clin Psychiatry. 2020;81(3).

133. Cosic K, Popovic S, Sarlija M, Kesedzic I. Impact of Human Disasters and COVID-19 Pandemic on Mental Health: Potential of Digital Psychiatry. Psychiatr Danub. 2020;32(1):25-31.

134. Crawford A, Serhal E. Digital Health Equity and COVID-19: The Innovation Curve Cannot Reinforce the Social Gradient of Health. J Med Internet Res. 2020;22(6):e19361.

135. Crowe M, Inder M, Farmar R, Carlyle D. Delivering psychotherapy by video conference in the time of COVID-19: Some considerations. J Psychiatr Ment Health Nurs. 2020.

136. Crump WJ. Telemedicine: Has the Time Really Finally Arrived? J Rural Health. 2020.

137. Cubo E, Hassan A, Bloem BR, Mari Z, Group MD-TS. Implementation of Telemedicine for Urgent and Ongoing Healthcare for Patients with Parkinson's Disease During the COVID-19 Pandemic: New Expectations for the Future. J Parkinsons Dis. 2020;10(3):911-3.

138. D'Amario D, Restivo A, Canonico F, Rodolico D, Mattia G, Francesco B, et al. Experience of remote cardiac care during the COVID-19 pandemic: the V-LAP device in advanced heart failure. Eur J Heart Fail. 2020;22(6):1050-2.

139. Daggubati LC, Eichberg DG, Ivan ME, Hanft S, Mansouri A, Komotar RJ, et al. Telemedicine for Outpatient Neurosurgical Oncology Care: Lessons Learned for the Future During the COVID-19 Pandemic. World Neurosurg. 2020;139:e859-e63.

140. Dantas LO, Barreto RPG, Ferreira CHJ. Digital physical therapy in the COVID-19 pandemic. Braz J Phys Ther. 2020;24(5):381-3.

141. Daruich A, Martin D, Bremond-Gignac D. Ocular manifestation as first sign of Coronavirus Disease 2019 (COVID-19): Interest of telemedicine during the pandemic context. J Fr Ophtalmol. 2020;43(5):389-91.

142. Das AV, Rani PK, Vaddavalli PK. Tele-consultations and electronic medical records driven remote patient care: Responding to the COVID-19 lockdown in India. Indian J Ophthalmol. 2020;68(6):1007-12.

143. Dasenbrook E. Keep cystic fibrosis patients out of the hospital. Cleve Clin J Med. 2020.

144. Dashraath P, Wong JLJ, Lim MXK, Lim LM, Li S, Biswas A, et al. Coronavirus disease 2019 (COVID-19) pandemic and pregnancy. Am J Obstet Gynecol. 2020;222(6):521-31.

145. Davarpanah AH, Mahdavi A, Sabri A, Langroudi TF, Kahkouee S, Haseli S, et al. Novel Screening and Triage Strategy in Iran During Deadly Coronavirus Disease 2019 (COVID-19) Epidemic: Value of Humanitarian Teleconsultation Service. J Am Coll Radiol. 2020;17(6):734-8.

146. de Azambuja E, Trapani D, Loibl S, Delaloge S, Senkus E, Criscitiello C, et al. ESMO Management and treatment adapted recommendations in the COVID-19 era: Breast Cancer. ESMO Open. 2020;5(Suppl 3).

147. de Girolamo G, Cerveri G, Clerici M, Monzani E, Spinogatti F, Starace F, et al. Mental Health in the Coronavirus Disease 2019 Emergency-The Italian Response. JAMA Psychiatry. 2020;77(9):974-6.

148. De Marchi F, Cantello R, Ambrosini S, Mazzini L, Group CS. Telemedicine and technological devices for amyotrophic lateral sclerosis in the era of COVID-19. Neurol Sci. 2020;41(6):1365-7.

149. de Marinis F, Attili I, Morganti S, Stati V, Spitaleri G, Gianoncelli L, et al. Results of Multilevel Containment Measures to Better Protect Lung Cancer Patients From COVID-19: The IEO Model. Front Oncol. 2020;10:665.

150. Dedeilia A, Sotiropoulos MG, Hanrahan JG, Janga D, Dedeilias P, Sideris M. Medical and Surgical Education Challenges and Innovations in the COVID-19 Era: A Systematic Review. In Vivo. 2020;34(3 Suppl):1603-11.

151. Denadai R. COVID-19 Pandemic as a Driver for Spreading Virtual Care Globally: The Future Starts Now. Clinics (Sao Paulo). 2020;75:e1967.

152. Denadai R, Lo LJ. Teleconsultation-mediated nasoalveolar molding therapy for babies with cleft lip/palate during the COVID-19 outbreak: Implementing change at pandemic speed. J Plast Reconstr Aesthet Surg. 2020;73(7):1357-404.

153. Denis F, Galmiche S, Dinh A, Fontanet A, Scherpereel A, Benezit F, et al. Epidemiological Observations on the Association Between Anosmia and COVID-19 Infection: Analysis of Data From a Self-Assessment Web Application. J Med Internet Res. 2020;22(6):e19855.

154. Dewar S, Lee PG, Suh TT, Min L. Uptake of Virtual Visits in A Geriatric Primary Care Clinic During the COVID-19 Pandemic. J Am Geriatr Soc. 2020;68(7):1392-4.

155. Dharmarajan H, Anderson JL, Kim S, Sridharan S, Duvvuri U, Ferris RL, et al. Transition to a virtual multidisciplinary tumor board during the COVID-19 pandemic: University of Pittsburgh experience. Head Neck. 2020;42(6):1310-6.

156. DiGiovanni G, Mousaw K, Lloyd T, Dukelow N, Fitzgerald B, D'Aurizio H, et al. Development of a telehealth geriatric assessment model in response to the COVID-19 pandemic. J Geriatr Oncol. 2020;11(5):761-3.

157. Ding XR, Clifton D, Ji N, Lovell NH, Bonato P, Chen W, et al. Wearable Sensing and Telehealth Technology with Potential Applications in the Coronavirus Pandemic. IEEE Rev Biomed Eng. 2020;PP.

158. Doshi A, Platt Y, Dressen JR, Mathews BK, Siy JC. Keep Calm and Log On: Telemedicine for COVID-19 Pandemic Response. J Hosp Med. 2020;15(5):302-4.

159. Dourado I, Magno L, Soares F, Massa P, Nunn A, Dalal S, et al. Adapting to the COVID-19 Pandemic: Continuing HIV Prevention Services for Adolescents Through Telemonitoring, Brazil. AIDS Behav. 2020;24(7):1994-9.

160. Duckett S. What should primary care look like after the COVID-19 pandemic? Aust J Prim Health. 2020;26(3):207-11.

161. Dumusc A, Dan D. [Rheumatology and COVID-19]. Rev Med Suisse. 2020;16(N degrees 691-2):831-4.

162. Dunnigan A. Letter to the Editor: In response to "Role of Telehealth in the Management of COVID-19: Lessons Learned from Previous SARS, MERS, and Ebola Outbreaks". Telemed J E Health. 2020;26(10):1209-10.

163. Eberly LA, Khatana SAM, Nathan AS, Snider C, Julien HM, Deleener ME, et al. Telemedicine Outpatient Cardiovascular Care during the COVID-19 Pandemic: Bridging or Opening the Digital Divide? Circulation. 2020.

164. Eccleston C, Blyth FM, Dear BF, Fisher EA, Keefe FJ, Lynch ME, et al. Managing patients with chronic pain during the COVID-19 outbreak: considerations for the rapid introduction of remotely supported (eHealth) pain management services. Pain. 2020;161(5):889-93.

165. Edelman LS, McConnell ES, Kennerly SM, Alderden J, Horn SD, Yap TL. Mitigating the Effects of a Pandemic: Facilitating Improved Nursing Home Care Delivery Through Technology. JMIR Aging. 2020;3(1):e20110.

166. Eghtesadi M. Breaking Social Isolation Amidst COVID-19: A Viewpoint on Improving Access to Technology in Long-Term Care Facilities. J Am Geriatr Soc. 2020;68(5):949-50.

167. Elbeddini A, Prabaharan T, Almasalkhi S, Tran C, Zhou Y. Barriers to conducting deprescribing in the elderly population amid the COVID-19 pandemic. Res Social Adm Pharm. 2020.

168. Elbeddini A, Yeats A. Pharmacist intervention amid the coronavirus disease 2019 (COVID-19) pandemic: from direct patient care to telemedicine. J Pharm Policy Pract. 2020;13:23.

169. Elkaddoum R, Haddad FG, Eid R, Kourie HR. Telemedicine for cancer patients during COVID-19 pandemic: between threats and opportunities. Future Oncol. 2020;16(18):1225-7.

170. Elkbuli A, Ehrlich H, McKenney M. The effective use of telemedicine to save lives and maintain structure in a healthcare system: Current response to COVID-19. Am J Emerg Med. 2020.

171. Ellis K, Lindley LC. A Virtual Children's Hospice in Response to COVID-19: The Scottish Experience. J Pain Symptom Manage. 2020;60(2):e40-e3.

172. Elmas OF, Demirbas A, Atasoy M, Tursen U, Lotti T. Teledermatology during COVID-19 pandemic: Ethical and legal considerations about the principles of treatment prescription and privacy. Dermatol Ther. 2020:e13781.

173. Elson EC, Oermann C, Duehlmeyer S, Bledsoe S. Use of telemedicine to provide clinical pharmacy services during the SARS-CoV-2 pandemic. Am J Health Syst Pharm. 2020;77(13):1005-6.

174. Emmanouil P, Karl P, Eleni R, Eleni K, Elias P, Constantinos T, et al. In the midst of the perfect storm: Swift public health actions needed in order to increase societal safety during the COVID-19 pandemic. Safety Science. 2020;129:104810.

175. Erwin C, Aultman J, Harter T, Illes J, Kogan RCJ. Rural and Remote Communities: Unique Ethical Issues in the COVID-19 Pandemic. Am J Bioeth. 2020;20(7):117-20.

176. Espinoza J, Crown K, Kulkarni O. A Guide to Chatbots for COVID-19 Screening at Pediatric Health Care Facilities. JMIR Public Health Surveill. 2020;6(2):e18808.

177. Evans DJR, Bay BH, Wilson TD, Smith CF, Lachman N, Pawlina W. Going Virtual to Support Anatomy Education: A STOPGAP in the Midst of the Covid-19 Pandemic. Anat Sci Educ. 2020;13(3):279-83.

178. Fagherazzi G, Goetzinger C, Rashid MA, Aguayo GA, Huiart L. Digital Health Strategies to Fight COVID-19 Worldwide: Challenges, Recommendations, and a Call for Papers. J Med Internet Res. 2020;22(6):e19284.

179. Fankhauser GT. Delivering high-quality vascular care by telehealth during the COVID-19 pandemic. J Vasc Surg. 2020;72(1):6-7.

180. Fantz CR, Rivers M. COVID-19 Awakens a New Focus on Surge Capacity Blood Glucose Testing and the Critical Role of Telehealth in Self-Management. J Diabetes Sci Technol. 2020;14(4):733-4.

181. Feng W, Zhang LN, Li JY, Wei T, Peng TT, Zhang DX, et al. [Analysis of special ehealth service for corona virus disease 2019 (COVID-19) pneumonia]. Beijing Da Xue Xue Bao Yi Xue Ban. 2020;52(2):302-7.

182. Fernandes L, FitzPatrick ME, Roycroft M. The role of the future physician: building on shifting sands. Clin Med (Lond). 2020.

183. Ferretti L, Wymant C, Kendall M, Zhao L, Nurtay A, Abeler-Dorner L, et al. Quantifying SARS-CoV-2 transmission suggests epidemic control with digital contact tracing. Science. 2020;368(6491).

184. Fisk M, Livingstone A, Pit SW. Telehealth in the Context of COVID-19: Changing Perspectives in Australia, the United Kingdom, and the United States. J Med Internet Res. 2020;22(6):e19264.

185. Fix OK, Serper M. Telemedicine and Telehepatology During the COVID-19 Pandemic. Clin Liver Dis (Hoboken). 2020;15(5):187-90.

186. Flint L, Kotwal A. The New Normal: Key Considerations for Effective Serious Illness Communication Over Video or Telephone During the Coronavirus Disease 2019 (COVID-19) Pandemic. Ann Intern Med. 2020;173(6):486-8.

187. Forbes RC, Solorzano CC, Concepcion BP. Surgical telemedicine here to stay: More support from a randomized controlled trial on postoperative surgery visits. Am J Surg. 2020;219(6):880-1.

188. Formigo-Couceiro J, Juan-Garcia FJ, Alonso-Bidegain M. [Coronavirus disease 2019 crisis. The challenge to take the final step to telerehabilitation]. Rehabilitacion (Madr). 2020;54(4):234-5.

189. Foulkes M. COVID-19 and cancer care. Br J Nurs. 2020;29(10):S3.

190. Franchi T. The Impact of the Covid-19 Pandemic on Current Anatomy Education and Future Careers: A Student's Perspective. Anat Sci Educ. 2020;13(3):312-5.

191. Freeman MP. COVID-19 From a Psychiatry Perspective: Meeting the Challenges. J Clin Psychiatry. 2020;81(2).

192. French JA, Brodie MJ, Caraballo R, Devinsky O, Ding D, Jehi L, et al. Keeping people with epilepsy safe during the COVID-19 pandemic. Neurology. 2020;94(23):1032-7.

193. Fritz MA, Howell RJ, Brodsky MB, Suiter DM, Dhar SI, Rameau A, et al. Moving Forward with Dysphagia Care: Implementing Strategies during the COVID-19 Pandemic and Beyond. Dysphagia. 2020.

194. Fuchs J, Hovorka R. COVID-19 and Diabetes: Could Diabetes Technology Research Help Pave the Way for Remote Healthcare? J Diabetes Sci Technol. 2020;14(4):735-6.

195. Gabriels J, Saleh M, Chang D, Epstein LM. Inpatient use of mobile continuous telemetry for COVID-19 patients treated with hydroxychloroquine and azithromycin. HeartRhythm Case Rep. 2020;6(5):241-3.

196. Gadzinski AJ, Andino JJ, Odisho AY, Watts KL, Gore JL, Ellimoottil C. Telemedicine and eConsults for Hospitalized Patients During COVID-19. Urology. 2020;141:12-4.

197. Gadzinski AJ, Ellimoottil C. Telehealth in urology after the COVID-19 pandemic. Nat Rev Urol. 2020;17(7):363-4.

198. Gadzinski AJ, Gore JL, Ellimoottil C, Odisho AY, Watts KL. Implementing Telemedicine in Response to the COVID-19 Pandemic. J Urol. 2020;204(1):14-6.

199. Galea-Singer S, Newcombe D, Farnsworth-Grodd V, Sheridan J, Adams P, Walker N. Challenges of virtual talking therapies for substance misuse in New Zealand during the COVID-19 pandemic: an opinion piece. N Z Med J. 2020;133(1515):104-11.

200. Gallagher RM. Pain Medicine Goes Digital: A Lamentation on COVID-19, the Printing Press, and the 21st Century. Pain Med. 2020.

201. Gamble A, Pham Q, Goyal S, Cafazzo JA. The Challenges of COVID-19 for People Living With Diabetes: Considerations for Digital Health. JMIR Diabetes. 2020;5(2):e19581.

202. Gan K, Liu Y, Stagg B, Rathi S, Pasquale LR, Damji K. Telemedicine for Glaucoma: Guidelines and Recommendations. Telemed J E Health. 2020;26(4):551-5.

203. Gao Z, Wang X, Huang X, Zhang J, Qu J. Development of a remote dedicated doctor platform of ophthalmology and its application efficiency during epidemic of COVID-19. Zhonghua Shiyan Yanke Zazhi/Chinese Journal of Experimental Ophthalmology. 2020;38(4):305-10.

204. Garg D, Dhamija RK. The Challenge of Managing Parkinson's Disease Patients during the COVID-19 Pandemic. Ann Indian Acad Neurol. 2020;23(Suppl 1):S24-S7.

205. Garg S, Bhatnagar N, Gangadharan N. A Case for Participatory Disease Surveillance of the COVID-19 Pandemic in India. JMIR Public Health Surveill. 2020;6(2):e18795.

206. Garg SK, Rodbard D, Hirsch IB, Forlenza GP. Managing New-Onset Type 1 Diabetes During the COVID-19 Pandemic: Challenges and Opportunities. Diabetes Technol Ther. 2020;22(6):431-9.

207. Georgakopoulou EA. Digitally aided telemedicine during the SARS-CoV-2 pandemic to screen oral medicine emergencies. Oral Dis. 2020.

208. Ghosh A, Gupta R, Misra A. Telemedicine for diabetes care in India during COVID19 pandemic and national lockdown period: Guidelines for physicians. Diabetes Metab Syndr. 2020;14(4):273-6.

209. Giansanti D, Aprile I. Letter to the Editor: Is the COVID-19 Pandemic an Opportunity to Enlarge the Telemedicine Boundaries? Telemed J E Health. 2020;26(9):1123-5.

210. Gibson A, Bardach SH, Pope ND. COVID-19 and the Digital Divide: Will Social Workers Help Bridge the Gap? J Gerontol Soc Work. 2020:1-3.

211. Gilbert AW, Billany JCT, Adam R, Martin L, Tobin R, Bagdai S, et al. Rapid implementation of virtual clinics due to COVID-19: report and early evaluation of a quality improvement initiative. BMJ Open Qual. 2020;9(2).

212. Glauser G, Wathen C, Miranda SP, Blue R, Dimentberg R, Welch WC, et al. Letter to the Editor Regarding "Implementation and Workflow of a Telehealth Clinic in Neurosurgery During the COVID-19 Pandemic". World Neurosurg. 2020;139:373-5.

213. Goh P-S, Sandars J. A vision of the use of technology in medical education after the COVID-19 pandemic. MedEdPublish. 2020;9.

214. Goldust M, Shivakumar S, Kroumpouzos G, Murrell DF, Rudnicka L, Jafferany M, et al. Virtual conferences of dermatology during the COVID-19 pandemic. Dermatol Ther. 2020:e13774.

215. Gondal KM, Shaukat S. Telemedicine in the Time of COVID-19 Pandemic. J Coll Physicians Surg Pak. 2020;30(4):349-50.

216. Gong K, Xu Z, Cai Z, Chen Y, Wang Z. Internet Hospitals Help Prevent and Control the Epidemic of COVID-19 in China: Multicenter User Profiling Study. J Med Internet Res. 2020;22(4):e18908.

217. Gonzales-Zamora JA, Alave J, De Lima-Corvino DF, Fernandez A. Videoconferences of Infectious Diseases: An educational tool that transcends borders. A useful tool also for the current COVID-19 pandemic. Infez Med. 2020;28(2):135-8.

218. Gonzalez-Perez R, Sanchez-Machin I, Poza-Guedes P, Matheu V, Alava-Cruz C, Mederos Luis E. Pertinence of Telehealth in a Rush Conversion to Virtual Allergy Practice during the COVID-19 Outbreak. J Investig Allergol Clin Immunol. 2020:0.

219. Goodman-Casanova JM, Dura-Perez E, Guzman-Parra J, Cuesta-Vargas A, Mayoral-Cleries F. Telehealth Home Support During COVID-19 Confinement for Community-Dwelling Older Adults With Mild Cognitive Impairment or Mild Dementia: Survey Study. J Med Internet Res. 2020;22(5):e19434.

220. Gorodeski EZ, Goyal P, Cox ZL, Thibodeau JT, Reay RE, Rasmusson K, et al. Virtual Visits for Care of Patients with Heart Failure in the Era of COVID-19: A Statement from the Heart Failure Society of America. J Card Fail. 2020;26(6):448-56.

221. Gould CE, Hantke NC. Promoting Technology and Virtual Visits to Improve Older Adult Mental Health in the Face of COVID-19. Am J Geriatr Psychiatry. 2020;28(8):889-90.

222. Govindarajan R, Berry JD, Paganoni S, Pulley MT, Simmons Z. Optimizing telemedicine to facilitate amyotrophic lateral sclerosis clinical trials. Muscle Nerve. 2020;62(3):321-6.

223. Goyal M, Ospel JM, Southerland AM, Wira C, Amin-Hanjani S, Fraser JF, et al. Prehospital Triage of Acute Stroke Patients During the COVID-19 Pandemic. Stroke. 2020;51(7):2263-7.

224. Graves JM, Mackelprang JL, Amiri S, Abshire DA. Barriers to Telemedicine Implementation in Southwest Tribal Communities During COVID-19. J Rural Health. 2020.

225. Greenhalgh T, Koh GCH, Car J. Covid-19: a remote assessment in primary care. BMJ. 2020;368:m1182.

226. Greenhalgh T, Wherton J, Shaw S, Morrison C. Video consultations for covid-19. BMJ. 2020;368:m998.

227. Greiwe J, Nyenhuis SM. Wearable Technology and How This Can Be Implemented into Clinical Practice. Curr Allergy Asthma Rep. 2020;20(8):36.

228. Grenda TR, Whang S, Evans NR, 3rd. Transitioning a Surgery Practice to Telehealth During COVID-19. Ann Surg. 2020;272(2):e168-e9.

229. Greven ACM, Rich CW, Malcolm JG, Bray DP, Rodts GE, Refai D, et al. Letter: Neurosurgical Management of Spinal Pathology Via Telemedicine During the COVID-19 Pandemic: Early Experience and Unique Challenges. Neurosurgery. 2020;87(2):E192-E6.

230. Grimes CL, Balk EM, Crisp CC, Antosh DD, Murphy M, Halder GE, et al. A guide for urogynecologic patient care utilizing telemedicine during the COVID-19 pandemic: review of existing evidence. Int Urogynecol J. 2020;31(6):1063-89.

231. Grossman SN, Han SC, Balcer LJ, Kurzweil A, Weinberg H, Galetta SL, et al. Rapid implementation of virtual neurology in response to the COVID-19 pandemic. Neurology. 2020;94(24):1077-87.

232. Gupta R, Ibraheim MK, Doan HQ. Teledermatology in the wake of COVID-19: Advantages and challenges to continued care in a time of disarray. J Am Acad Dermatol. 2020;83(1):168-9.

233. Gutierrez J, Kuperman E, Kaboli PJ. Using Telehealth as a Tool for Rural Hospitals in the COVID-19 Pandemic Response. J Rural Health. 2020.

234. Gyorffy Z, Bekasi S, Szathmari-Meszaros N, Nemeth O. Possibilities of telemedicine regarding the COVID-19 pandemic in light of the international and Hungarian experiences and recommendations. Orv Hetil. 2020;161(24):983-92.

235. Hagge D, Knopf A, Hofauer B. [Telemedicine in the fight against SARS-COV-2-opportunities and possible applications in otorhinolaryngology : Narrative review]. HNO. 2020;68(6):433-9.

236. Hakim AA, Kellish AS, Atabek U, Spitz FR, Hong YK. Implications for the use of telehealth in surgical patients during the COVID-19 pandemic. Am J Surg. 2020;220(1):48-9.

237. Hamza M, Khan HS, Sattar ZA, Hanif M. Doctor-patient communication in surgical practice during the coronavirus (COVID-19) pandemic. Br J Surg. 2020;107(7):e193.

238. Hanel E, Bilic M, Hassall K, Hastings M, Jazuli F, Ha M, et al. Virtual application of in situ simulation during a pandemic. CJEM. 2020:1-6.

239. Hannon P, Lappe K, Griffin C, Roussel D, Colbert-Getz J. An objective structured clinical examination: From examination room to Zoom breakout room. Med Educ. 2020;54(9):861.

240. Harris M, Johnson S, Mackin S, Saitz R, Walley AY, Taylor JL. Low Barrier Tele-Buprenorphine in the Time of COVID-19: A Case Report. J Addict Med. 2020;14(4):e136-e8.

241. Hau YS, Kim JK, Hur J, Chang MC. How about actively using telemedicine during the COVID-19 pandemic? J Med Syst. 2020;44(6):108.

242. Hernandez Benabe S, Langshaw AH. IBD in the Times of COVID-19. Inflamm Bowel Dis. 2020;26(8):e92.

243. Hernando-Requejo V, Huertas-Gonzalez N, Lapena-Motilva J, Ogando-Duran G. The epilepsy unit during the COVID-19 epidemic: The role of telemedicine and the effects of confinement on patients with epilepsy. Neurologia. 2020;35(4):274-6.

244. Higgins PDR, Ng S, Danese S, Rao K. The Risk of SARS-CoV-2 in Immunosuppressed IBD Patients. Crohns Colitis 360. 2020;2(2):otaa026.

245. Hightow-Weidman L, Muessig K, Claude K, Roberts J, Zlotorzynska M, Sanchez T. Maximizing Digital Interventions for Youth in the Midst of Covid-19: Lessons from the Adolescent Trials Network for HIV Interventions. AIDS Behav. 2020;24(8):2239-43.

246. Hoagland B, Torres TS, Bezerra DRB, Geraldo K, Pimenta C, Veloso VG, et al. Telemedicine as a tool for PrEP delivery during the COVID-19 pandemic in a large HIV prevention service in Rio de Janeiro-Brazil. Braz J Infect Dis. 2020;24(4):360-4.

247. Hoffman GJ, Webster NJ, Bynum JPW. A Framework for Aging-Friendly Services and Supports in the Age of COVID-19. J Aging Soc Policy. 2020;32(4-5):450-9.

248. Hofmann H, Harding C, Youm J, Wiechmann W. Virtual bedside teaching rounds with patients with COVID-19. Med Educ. 2020.

249. Hollander JE, Carr BG. Virtually Perfect? Telemedicine for Covid-19. N Engl J Med. 2020;382(18):1679-81.

250. Holstead RG, Robinson AG. Discussing Serious News Remotely: Navigating Difficult Conversations During a Pandemic. JCO Oncol Pract. 2020;16(7):363-8.

251. Hong YR, Lawrence J, Williams D, Jr., Mainous IA. Population-Level Interest and Telehealth Capacity of US Hospitals in Response to COVID-19: Cross-Sectional Analysis of Google Search and National Hospital Survey Data. JMIR Public Health Surveill. 2020;6(2):e18961.

252. Hong Z, Li N, Li D, Li J, Li B, Xiong W, et al. Telemedicine During the COVID-19 Pandemic: Experiences From Western China. J Med Internet Res. 2020;22(5):e19577.

253. Hoopes S, Pham T, Lindo FM, Antosh DD. Home Surgical Skill Training Resources for Obstetrics and Gynecology Trainees During a Pandemic. Obstet Gynecol. 2020;136(1):56-64.

254. Hsieh MW, Lee CC, Ou SF, Kuo YR. Telemedicine algorithm for chronic wound care during COVID-19. Int Wound J. 2020.

255. Hsu TC, Wu CC, Lai PY, Syue LS, Lai YY, Ko NY. [Nursing Experience of Caring for a Patient With COVID-19 During Isolation]. Hu Li Za Zhi. 2020;67(3):111-9.

256. Huang S, Xiao Y, Yan L, Deng J, He M, Lu J, et al. Implications for Online Management: Two Cases with COVID-19. Telemed J E Health. 2020;26(4):487-94.

257. Humphreys J, Schoenherr L, Elia G, Saks NT, Brown C, Barbour S, et al. Rapid Implementation of Inpatient Telepalliative Medicine Consultations During COVID-19 Pandemic. J Pain Symptom Manage. 2020;60(1):e54-e9.

258. Inkster B, O'Brien R, Selby E, Joshi S, Subramanian V, Kadaba M, et al. Digital Health Management During and Beyond the COVID-19 Pandemic: Opportunities, Barriers, and Recommendations. JMIR Ment Health. 2020;7(7):e19246.

259. Israilov S, Krouss M, Zaurova M, Jalon HS, Conley G, Shulman P, et al. National Outreach of Telepalliative Medicine Volunteers for a New York City Safety Net System COVID-19 Pandemic Response. J Pain Symptom Manage. 2020;60(2):e14-e7.

260. Iyengar K, Upadhyaya GK, Vaishya R, Jain V. COVID-19 and applications of smartphone technology in the current pandemic. Diabetes Metab Syndr. 2020;14(5):733-7.

261. Iyengar K, Vaish A, Toh E, Vaishya R. COVID-19 and remote consulting strategies in managing trauma and orthopaedics. Postgrad Med J. 2020;96(1137):438-9.

262. Jakhar D, Kaul S, Kaur I. WhatsApp messenger as a teledermatology tool during coronavirus disease (COVID-19): from bedside to phone-side. Clin Exp Dermatol. 2020;45(6):739-40.

263. Jamil B. Clinical features, diagnosis and management of COVID-19 patients in the outdoor setting. J Pak Med Assoc. 2020;70(Suppl 3)(5):S52-S5.

264. Janda M, Swetter SM, Horsham C, Soyer HP. Virtual melanoma checks during a pandemic. Br J Dermatol. 2020;183(4):752-3.

265. Japan ECMOnet for COVID-19. Japan ECMOnet for COVID-19: Telephone consultations for cases with severe respiratory failure caused by COVID-19. Journal of Intensive Care. 2020;8(1):24.

266. Jayawardena ADL, Mankarious LA, Keamy DG, Jr., Cohen MS. Pediatric, Family-Centered, "At-Home" Otologic Physical Examination in the COVID-19 Era. Otolaryngol Head Neck Surg. 2020:194599820934776.

267. Jendle J. The Use of eHealth for the Care of Patients With Diabetes in Connection to the COVID-19 Pandemic. J Diabetes Sci Technol. 2020;14(4):739-40.

268. Jethwa T, Ton A, Paredes Molina CS, Speicher L, Walsh K, Knight D, et al. Establishing Mayo Clinic's Coronavirus Disease 2019 Virtual Clinic: A Preliminary Communication. Telemed J E Health. 2020.

269. John O. Video consultations for triage of patients with covid-19. BMJ. 2020;369:m1583.

270. Jones MS, Goley AL, Alexander BE, Keller SB, Caldwell MM, Buse JB. Inpatient Transition to Virtual Care During COVID-19 Pandemic. Diabetes Technol Ther. 2020;22(6):444-8.

271. Jordan A, Dixon LB. Considerations for Telepsychiatry Service Implementation in the Era of COVID-19. Psychiatr Serv. 2020;71(6):643-4.

272. Kanatas A, Rogers SN. The role of the Head and Neck cancer-specific Patient Concerns Inventory (PCI-HN) in telephone consultations during the COVID-19 pandemic. Br J Oral Maxillofac Surg. 2020;58(5):497-9.

273. Kang S, Thomas PBM, Sim DA, Parker RT, Daniel C, Uddin JM. Oculoplastic video-based telemedicine consultations: Covid-19 and beyond. Eye (Lond). 2020;34(7):1193-5.

274. Kannampallil T, Ma J. Digital Translucence: Adapting Telemedicine Delivery Post-COVID-19. Telemed J E Health. 2020;26(9):1120-2.

275. Kannarkat JT, Smith NN, McLeod-Bryant SA. Mobilization of Telepsychiatry in Response to COVID-19-Moving Toward 21(st) Century Access to Care. Adm Policy Ment Health. 2020;47(4):489-91.

276. Kanneganti A, Lim KMX, Chan GMF, Choo SN, Choolani M, Ismail-Pratt I, et al. Pedagogy in a pandemic - COVID-19 and virtual continuing medical education (vCME) in obstetrics and gynecology. Acta Obstet Gynecol Scand. 2020;99(6):692-5.

277. Kanneganti A, Sia CH, Ashokka B, Ooi SBS. Continuing medical education during a pandemic: an academic institution's experience. Postgrad Med J. 2020;96(1137):384-6.

278. Karanam S, Li R, Yang F, Hu W, Chen T, Wu Z. Towards Contactless Patient Positioning. IEEE Trans Med Imaging. 2020;39(8):2701-10.

279. Karim JS, Hachach-Haram N, Dasgupta P. Bolstering the surgical response to COVID-19: how virtual technology will save lives and safeguard surgical practice. BJU Int. 2020;125(6):E18-E9.

280. Kasle DA, Torabi SJ, Savoca EL, Judson BL, Manes RP. Outpatient Otolaryngology in the Era of COVID-19: A Data-Driven Analysis of Practice Patterns. Otolaryngol Head Neck Surg. 2020;163(1):138-44.

281. Katapally TR. A Global Digital Citizen Science Policy to Tackle Pandemics Like COVID-19. J Med Internet Res. 2020;22(5):e19357.

282. Katz JN, Sinha SS, Alviar CL, Dudzinski DM, Gage A, Brusca SB, et al. COVID-19 and Disruptive Modifications to Cardiac Critical Care Delivery: JACC Review Topic of the Week. J Am Coll Cardiol. 2020;76(1):72-84.

283. Kavoor AR, Chakravarthy K, John T. Remote consultations in the era of COVID-19 pandemic: Preliminary experience in a regional Australian public acute mental health care setting. Asian J Psychiatr. 2020;51:102074.

284. Keesara S, Jonas A, Schulman K. Covid-19 and Health Care's Digital Revolution. N Engl J Med. 2020;382(23):e82.

285. Kemp MT, Williams AM, Alam HB. eClinic: increasing use of telehealth as a risk reduction strategy during the covid-19 pandemic. Trauma Surg Acute Care Open. 2020;5(1):e000481.

286. Kerber AA, Soma DB, Youssef MJ. Chilblains-like dermatologic manifestation of COVID-19 diagnosed by serology via multidisciplinary virtual care. Int J Dermatol. 2020;59(8):1024-5.

287. Keshvardoost S, Bahaadinbeigy K, Fatehi F. Role of Telehealth in the Management of COVID-19: Lessons Learned from Previous SARS, MERS, and Ebola Outbreaks. Telemed J E Health. 2020;26(7):850-2.

288. Khairat S, Meng C, Xu Y, Edson B, Gianforcaro R. Interpreting COVID-19 and Virtual Care Trends: Cohort Study. JMIR Public Health Surveill. 2020;6(2):e18811.

289. Khan ZH, Siddique A, Lee CW. Robotics Utilization for Healthcare Digitization in Global COVID-19 Management. Int J Environ Res Public Health. 2020;17(11).

290. Khanna R, Forbes M. Telepsychiatry as a public health imperative: Slowing COVID-19. Aust N Z J Psychiatry. 2020;54(7):758.

291. Khullar G, Chandra M. Virtual dermatopathology: A potential educational tool during COVID-19 pandemic. Dermatol Ther. 2020:e13755.

292. Kim RH, Brinster NK, Meehan SA. Dermatopathology education during the COVID-19 pandemic: Virtual simulation of the multiheaded microscope. J Am Acad Dermatol. 2020;83(3):e243-e4.

293. Kim SW, Lee KS, Kim K, Lee JJ, Kim JY, Daegu Medical A. A Brief Telephone Severity Scoring System and Therapeutic Living Centers Solved Acute Hospital-Bed Shortage during the COVID-19 Outbreak in Daegu, Korea. J Korean Med Sci. 2020;35(15):e152.

294. Kirkpatrick AW, McKee JL. Lung ultrasonography in a woman with COVID-19: This examination could be remote. CMAJ. 2020;192(16):E435.

295. Klein BC, Busis NA. COVID-19 is catalyzing the adoption of teleneurology. Neurology. 2020;94(21):903-4.

296. Klonoff DC. Telemedicine for Diabetes After the COVID-19 Pandemic: We Can't Put the Toothpaste Back in the Tube or Turn Back the Clock. J Diabetes Sci Technol. 2020;14(4):741-2.

297. Klum M, Urban M, Tigges T, Pielmus AG, Feldheiser A, Schmitt T, et al. Wearable Cardiorespiratory Monitoring Employing a Multimodal Digital Patch Stethoscope: Estimation of ECG, PEP, LVETand Respiration Using a 55 mm Single-Lead ECG and Phonocardiogram. Sensors (Basel). 2020;20(7).

298. Kogan M, Klein SE, Hannon CP, Nolte MT. Orthopaedic Education During the COVID-19 Pandemic. J Am Acad Orthop Surg. 2020;28(11):e456-e64.

299. Koumpouras F, Helfgott S. Stand Together and Deliver: Challenges and Opportunities for Rheumatology Education During the COVID-19 Pandemic. Arthritis Rheumatol. 2020;72(7):1064-6.

300. Krausz M, Westenberg JN, Vigo D, Spence RT, Ramsey D. Emergency Response to COVID-19 in Canada: Platform Development and Implementation for eHealth in Crisis Management. JMIR Public Health Surveill. 2020;6(2):e18995.

301. Krukowski RA, Ross KM. Measuring Weight with Electronic Scales in Clinical and Research Settings During the Coronavirus Disease 2019 Pandemic. Obesity (Silver Spring). 2020;28(7):1182-3.

302. Kumar S, Bishnoi A, Vinay K. Changing paradigms of dermatology practice in developing nations in the shadow of COVID-19: Lessons learnt from the pandemic. Dermatol Ther. 2020:e13472.

303. Kwon R, Zhang ML, VandenBussche CJ. Considerations for remote learning in pathology during COVID-19 social distancing. Cancer Cytopathol. 2020;128(9):642-7.

304. Lam PW, Sehgal P, Andany N, Mubareka S, Simor AE, Ozaldin O, et al. A virtual care program for outpatients diagnosed with COVID-19: a feasibility study. CMAJ Open. 2020;8(2):E407-E13.

305. Latifi R, Doarn CR. Perspective on COVID-19: Finally, Telemedicine at Center Stage. Telemed J E Health. 2020;26(9):1106-9.

306. Launois S, Gagnadoux F, Arnulf I, Charley Monaca C, Dauvilliers Y, d'Ortho MP, et al. Proposals for the practice of sleep medicine during the COVID-19 pandemic. Medecine du Sommeil. 2020.

307. Layfield E, Triantafillou V, Prasad A, Deng J, Shanti RM, Newman JG, et al. Telemedicine for head and neck ambulatory visits during COVID-19: Evaluating usability and patient satisfaction. Head Neck. 2020;42(7):1681-9.

308. Lee AC. COVID-19 and the Advancement of Digital Physical Therapist Practice and Telehealth. Phys Ther. 2020;100(7):1054-7.

309. Lee AKF, Cho RHW, Lau EHL, Cheng HK, Wong EWY, Ku PKM, et al. Mitigation of head and neck cancer service disruption during COVID-19 in Hong Kong through telehealth and multi-institutional collaboration. Head Neck. 2020;42(7):1454-9.

310. Lee I, Kovarik C, Tejasvi T, Pizarro M, Lipoff JB. Telehealth: Helping your patients and practice survive and thrive during the COVID-19 crisis with rapid quality implementation. J Am Acad Dermatol. 2020;82(5):1213-4.

311. Lees CW, Regueiro M, Mahadevan U, International Organization for the Study of Inflammatory Bowel D. Innovation in Inflammatory Bowel Disease Care During the COVID-19 Pandemic: Results of a Global Telemedicine Survey by the International Organization for the Study of Inflammatory Bowel Disease. Gastroenterology. 2020;159(3):805-8 e1.

312. Lepkowsky CM. Telehealth Reimbursement Allows Access to Mental Health Care During COVID-19. Am J Geriatr Psychiatry. 2020;28(8):898-9.

313. Li CH, Rajamohan AG, Acharya PT, Liu CJ, Patel V, Go JL, et al. Virtual Read-Out: Radiology Education for the 21st Century During the COVID-19 Pandemic. Acad Radiol. 2020;27(6):872-81.

314. Li H, Zheng S, Liu F, Liu W, Zhao R. Fighting against COVID-19: Innovative strategies for clinical pharmacists. Res Social Adm Pharm. 2020.

315. Lin B, Wu S. COVID-19 (Coronavirus Disease 2019): Opportunities and Challenges for Digital Health and the Internet of Medical Things in China. OMICS. 2020;24(5):231-2.

316. Lincoln H, Khan R, Cai J. Telecommuting: A viable option for medical physicists amid the COVID-19 outbreak and beyond. Med Phys. 2020;47(5):2045-8.

317. Lindsay JA, Hogan JB, Ecker AH, Day SC, Chen P, Helm A. The Importance of Video Visits in the Time of COVID-19. J Rural Health. 2020.

318. Liu R, Sundaresan T, Reed ME, Trosman JR, Weldon CB, Kolevska T. Telehealth in Oncology During the COVID-19 Outbreak: Bringing the House Call Back Virtually. JCO Oncol Pract. 2020;16(6):289-93.

319. Liu S, Yang L, Zhang C, Xiang YT, Liu Z, Hu S, et al. Online mental health services in China during the COVID-19 outbreak. Lancet Psychiatry. 2020;7(4):e17-e8.

320. Liu X, Zhang D, Sun T, Li X, Zhang H. Containing COVID-19 in rural and remote areas: experiences from China. J Travel Med. 2020;27(3).

321. Loeb AE, Rao SS, Ficke JR, Morris CD, Riley LH, 3rd, Levin AS. Departmental Experience and Lessons Learned With Accelerated Introduction of Telemedicine During the COVID-19 Crisis. J Am Acad Orthop Surg. 2020;28(11):e469-e76.

322. Loh TY, Hsiao JL, Shi VY. COVID-19 and its effect on medical student education in dermatology. J Am Acad Dermatol. 2020;83(2):e163-e4.

323. Looi JC, Pring W. Private metropolitan telepsychiatry in Australia during Covid-19: current practice and future developments. Australas Psychiatry. 2020;28(5):508-10.

324. Lopes MA, Santos-Silva AR, Vargas PA, Kowalski LP. Virtual assistance in oral medicine for prioritizing oral cancer diagnosis during the COVID-19 pandemic. Oral Surg Oral Med Oral Pathol Oral Radiol. 2020;130(1):127-8.

325. Lopez-Medina C, Escudero A, Collantes-Estevez E. COVID-19 pandemic: an opportunity to assess the utility of telemedicine in patients with rheumatic diseases. Ann Rheum Dis. 2020.

326. Luciani LG, Mattevi D, Cai T, Giusti G, Proietti S, Malossini G. Teleurology in the Time of Covid-19 Pandemic: Here to Stay? Urology. 2020;140:4-6.

327. Machado RA, Bonan PRF, Perez D, Martelli DRB, Martelli-Junior H. I am having trouble keeping up with virtual teaching activities: Reflections in the COVID-19 era. Clinics (Sao Paulo). 2020;75:e1945.

328. Machado RA, de Souza NL, Oliveira RM, Martelli Junior H, Bonan PRF. Social media and telemedicine for oral diagnosis and counselling in the COVID-19 era. Oral Oncol. 2020;105:104685.

329. Madden N, Emeruwa UN, Friedman AM, Aubey JJ, Aziz A, Baptiste CD, et al. Telehealth Uptake into Prenatal Care and Provider Attitudes during the COVID-19 Pandemic in New York City: A Quantitative and Qualitative Analysis. Am J Perinatol. 2020;37(10):1005-14.

330. Mader JK. Personal Experiences With Coronavirus Disease 2019 and Diabetes: The Time for Telemedicine is Now. J Diabetes Sci Technol. 2020;14(4):752-3.

331. Madrigal E. Going remote: Maintaining normalcy in our pathology laboratories during the COVID-19 pandemic. Cancer Cytopathol. 2020;128(5):321-2.

332. Maeshima S, Tamiya T, Saeki T, Ohkawara M, Osakabe M, Take Y, et al. Remote Rehabilitation Conferences in the Age of SARS-CoV-2. Am J Phys Med Rehabil. 2020;99(9):783.

333. Maggio MG, De Luca R, Manuli A, Calabro RS. The five 'W' of cognitive telerehabilitation in the Covid-19 era. Expert Rev Med Devices. 2020;17(6):473-5.

334. Mahajan V, Singh T, Azad C. Using Telemedicine During the COVID-19 Pandemic. Indian Pediatr. 2020;57(7):652-7.

335. Mahmood S, Hasan K, Colder Carras M, Labrique A. Global Preparedness Against COVID-19: We Must Leverage the Power of Digital Health. JMIR Public Health Surveill. 2020;6(2):e18980.

336. Mahon SM. Telegenetics: Remote Counseling During the COVID-19 Pandemic. Clin J Oncol Nurs. 2020;24(3):244-8.

337. Makhni MC, Riew GJ, Sumathipala MG. Telemedicine in Orthopaedic Surgery: Challenges and Opportunities. J Bone Joint Surg Am. 2020;102(13):1109-15.

338. Mallon D, Pohl JF, Phatak UP, Fernandes M, Rosen JM, Lusman SS, et al. Impact of COVID-19 on Pediatric Gastroenterology Fellow Training in North America. J Pediatr Gastroenterol Nutr. 2020;71(1):6-11.

339. Manjunatha N, Kumar CN, Math SB. Coronavirus disease 2019 pandemic: Time to optimize the potential of telepsychiatric aftercare clinic to ensure the continuity of care. Indian J Psychiatry. 2020;62(3):320-1.

340. Mann DM, Chen J, Chunara R, Testa PA, Nov O. COVID-19 transforms health care through telemedicine: Evidence from the field. J Am Med Inform Assoc. 2020;27(7):1132-5.

341. Marasca C, Ruggiero A, Fontanella G, Ferrillo M, Fabbrocini G, Villani A. Telemedicine and support groups could be used to improve adherence to treatment and

health-related quality of life in patients affected by inflammatory skin conditions during the COVID-19 pandemic. Clin Exp Dermatol. 2020;45(6):749.

342. Maret D, Peters OA, Vaysse F, Vigarios E. Integration of telemedicine into the public health response to COVID-19 must include dentists. Int Endod J. 2020;53(6):880-1.

343. Mariani S, Hanke JS, Dogan G, Schmitto JD. Out of hospital management of LVAD patients during COVID-19 outbreak. Artif Organs. 2020;44(8):873-6.

344. Marques da Silva B. Will Virtual Teaching Continue After the COVID-19 Pandemic? Acta Med Port. 2020;33(6):446.

345. Marshall JM, Dunstan DA, Bartik W. The role of digital mental health resources to treat trauma symptoms in Australia during COVID-19. Psychol Trauma. 2020;12(S1):S269-S71.

346. Matalon SA, Souza DAT, Gaviola GC, Silverman SG, Mayo-Smith WW, Lee LK. Trainee and Attending Perspectives on Remote Radiology Readouts in the Era of the COVID-19 Pandemic. Acad Radiol. 2020;27(8):1147-53.

347. Matias-Guiu J, Matias-Guiu JA, Alvarez-Sabin J, Ramon Ara J, Arenillas J, Casado-Naranjo I, et al. Will neurological care change over the next 5 years due to the COVID-19 pandemic? Key informant consensus survey. Neurologia. 2020;35(4):252-7.

348. Mattei A, Amy de la Breteque B, Crestani S, Crevier-Buchman L, Galant C, Hans S, et al. Guidelines of clinical practice for the management of swallowing disorders and recent dysphonia in the context of the COVID-19 pandemic. Eur Ann Otorhinolaryngol Head Neck Dis. 2020;137(3):173-5.

349. Matthewson J, Tiplady A, Gerakios F, Foley A, Murphy E. Implementation and analysis of a telephone support service during COVID-19. Occup Med (Lond). 2020;70(5):375-81.

350. Maurrasse SE, Rastatter JC, Hoff SR, Billings KR, Valika TS. Telemedicine During the COVID-19 Pandemic: A Pediatric Otolaryngology Perspective. Otolaryngol Head Neck Surg. 2020;163(3):480-1.

351. McDowell L, Goode S, Sundaresan P. Adapting to a global pandemic through live virtual delivery of a cancer collaborative trial group conference: The TROG 2020 experience. J Med Imaging Radiat Oncol. 2020;64(3):414-21.

352. McGrath J. ADHD and Covid-19: current roadblocks and future opportunities. Ir J Psychol Med. 2020;37(3):204-11.

353. McKechnie T, Levin M, Zhou K, Freedman B, Palter VN, Grantcharov TP. Virtual Surgical Training During COVID-19: Operating Room Simulation Platforms Accessible From Home. Ann Surg. 2020;272(2):e153-e4.

354. Medina M, Babiuch C, Card M, Gavrilescu R, Zafirau W, Boose E, et al. Home monitoring for COVID-19. Cleve Clin J Med. 2020.

355. Medina-Walpole A. In COVID-19 response to Congress & Administration, AGS calls for access to medical supplies, telehealth, among other needs. Geriatr Nurs. 2020;41(3):347-8.

356. Mehrotra A, Ray K, Brockmeyer DM, Barnett ML, Bender JA. Rapidly converting to “virtual practices”: outpatient care in the era of Covid-19. NEJM catalyst innovations in care delivery. 2020;1(2).

357. Meloni M, Izzo V, Giurato L, Gandini R, Uccioli L. Management of diabetic persons with foot ulceration during COVID-19 health care emergency: Effectiveness of a new triage pathway. Diabetes Res Clin Pract. 2020;165:108245.

358. Meng X, Dai Z, Hang C, Wang Y. Smartphone-enabled wireless otoscope-assisted online telemedicine during the COVID-19 outbreak. Am J Otolaryngol. 2020;41(3):102476.

359. Meti N, Rossos PG, Cheung MC, Singh S. Virtual Cancer Care During and Beyond the COVID-19 Pandemic: We Need to Get It Right. JCO Oncol Pract. 2020;16(9):527-9.

360. MF OR, Merghani K, Sheehan E. Virtualised care and COVID-19. Ir J Med Sci. 2020.

361. Mgbako O, Miller EH, Santoro AF, Remien RH, Shalev N, Olender S, et al. COVID-19, Telemedicine, and Patient Empowerment in HIV Care and Research. AIDS Behav. 2020;24(7):1990-3.

362. Mian A, Khan S. Medical education during pandemics: a UK perspective. BMC Med. 2020;18(1):100.

363. Michaud L, Stiefel F, Gasser J. [Psychiatry in the time of pandemic : forging new ways without getting lost]. Rev Med Suisse. 2020;16(N degrees 691-2):855-8.

364. Middleton A, Simpson KN, Bettger JP, Bowden MG. COVID-19 Pandemic and Beyond: Considerations and Costs of Telehealth Exercise Programs for Older Adults With Functional Impairments Living at Home-Lessons Learned From a Pilot Case Study. Phys Ther. 2020;100(8):1278-88.

365. Minniti A, Maglione W, Pignataro F, Cappadona C, Caporali R, Del Papa N. Taking care of systemic sclerosis patients during COVID-19 pandemic: rethink the clinical activity. Clin Rheumatol. 2020;39(7):2063-5.

366. Mishra V. Factors affecting the adoption of telemedicine during COVID-19. Indian J Public Health. 2020;64(Supplement):S234-S6.

367. Moazzami B, Razavi-Khorasani N, Dooghaie Moghadam A, Farokhi E, Rezaei N. COVID-19 and telemedicine: Immediate action required for maintaining healthcare providers well-being. J Clin Virol. 2020;126:104345.

368. Moccia M, Lanzillo R, Brescia Morra V, Bonavita S, Tedeschi G, Leocani L, et al. Assessing disability and relapses in multiple sclerosis on tele-neurology. Neurol Sci. 2020;41(6):1369-71.

369. Montesi G, Di Biase S, Chierchini S, Pavanato G, Virdis GE, Contato E, et al. Radiotherapy during COVID-19 pandemic. How to create a No fly zone: a Northern Italy experience. Radiol Med. 2020;125(6):600-3.

370. Mouchtouris N, Lavergne P, Montenegro TS, Gonzalez G, Baldassari M, Sharan A, et al. Telemedicine in Neurosurgery: Lessons Learned and Transformation of Care During the COVID-19 Pandemic. World Neurosurg. 2020;140:e387-e94.

371. Mukaino M, Tatemoto T, Kumazawa N, Tanabe S, Katoh M, Saitoh E, et al. Staying Active in Isolation: Telerehabilitation for Individuals With the Severe Acute Respiratory Syndrome Coronavirus 2 Infection. Am J Phys Med Rehabil. 2020;99(6):478-9.

372. Murdock HM, Penner JC, Le S, Nematollahi S. Virtual Morning Report during COVID-19: A novel model for case-based teaching conferences. Med Educ. 2020;54(9):851-2.

373. Murphy HR. Managing Diabetes in Pregnancy Before, During, and After COVID-19. Diabetes Technol Ther. 2020;22(6):454-61.

374. Myers US, Birks A, Grubaugh AL, Axon RN. Flattening the Curve by Getting Ahead of It: How the VA Healthcare System Is Leveraging Telehealth to Provide Continued Access to Care for Rural Veterans. J Rural Health. 2020.

375. Nadgir R. Teaching Remotely: Educating Radiology Trainees at the Workstation in the COVID-19 Era. Acad Radiol. 2020;27(9):1291-3.

376. Nagata JM. Rapid Scale-Up of Telehealth During the COVID-19 Pandemic and Implications for Subspecialty Care in Rural Areas. J Rural Health. 2020.

377. Nagra M, Vianya-Estopa M, Wolffsohn JS. Could telehealth help eye care practitioners adapt contact lens services during the COVID-19 pandemic? Cont Lens Anterior Eye. 2020;43(3):204-7.

378. Navarrete-Reyes AP, Avila-Funes JA. Staying in a Burning House: Perks and Perils of a Hotline in the Times of COVID-19. J Am Geriatr Soc. 2020;68(5):E10-E1.

379. Negrini S, Kiekens C, Bernetti A, Capecci M, Ceravolo MG, Lavezzi S, et al. Telemedicine from research to practice during the pandemic. "Instant paper from the field" on rehabilitation answers to the COVID-19 emergency. Eur J Phys Rehabil Med. 2020;56(3):327-30.

380. Neubeck L, Hansen T, Jaarsma T, Klompstra L, Gallagher R. Delivering healthcare remotely to cardiovascular patients during COVID-19 : A rapid review of the evidence. Eur J Cardiovasc Nurs. 2020;19(6):486-94.

381. Newell-Price J, Nieman LK, Reincke M, Tabarin A. ENDOCRINOLOGY IN THE TIME OF COVID-19: Management of Cushing's syndrome. Eur J Endocrinol. 2020;183(1):G1-G7.

382. Newhouse N, Farmer A, Whelan ME. COVID-19: Needs-led implementation and the immediate potential of remote monitoring. BJGP Open. 2020;4(2).

383. Nguyen NP, Vinh-Hung V, Baumert B, Zamagni A, Arenas M, Motta M, et al. Older Cancer Patients during the COVID-19 Epidemic: Practice Proposal of the International Geriatric Radiotherapy Group. Cancers (Basel). 2020;12(5).

384. Nitkunan A, Paviour D, Nitkunan T. COVID-19: switching to remote neurology outpatient consultations. Pract Neurol. 2020;20(3):222-4.

385. Nittas V, von Wyl V. COVID-19 and telehealth: a window of opportunity and its challenges. Swiss Med Wkly. 2020;150:w20284.

386. Nogueira MS. Biophotonic telemedicine for disease diagnosis and monitoring during pandemics: Overcoming COVID-19 and shaping the future of healthcare. Photodiagnosis Photodyn Ther. 2020;31:101836.

387. Noone C, McSharry J, Smalle M, Burns A, Dwan K, Devane D, et al. Video calls for reducing social isolation and loneliness in older people: a rapid review. Cochrane Database Syst Rev. 2020;5:CD013632.

388. Norgaard K. Telemedicine Consultations and Diabetes Technology During COVID-19. J Diabetes Sci Technol. 2020;14(4):767-8.

389. Noureldine MHA, Pressman E, Krafft PR, Greenberg MS, Agazzi S, van Loveren H, et al. Impact of the COVID-19 Pandemic on Neurosurgical Practice at an Academic Tertiary Referral Center: A Comparative Study. World Neurosurg. 2020;139:e872-e6.

390. O'Brien M, McNicholas F. The use of telepsychiatry during COVID-19 and beyond. Ir J Psychol Med. 2020:1-6.

391. Obeid JS, Davis M, Turner M, Meystre SM, Heider PM, O'Bryan EC, et al. An artificial intelligence approach to COVID-19 infection risk assessment in virtual visits: A case report. J Am Med Inform Assoc. 2020;27(8):1321-5.

392. Ohannessian R, Duong TA, Odone A. Global Telemedicine Implementation and Integration Within Health Systems to Fight the COVID-19 Pandemic: A Call to Action. JMIR Public Health Surveill. 2020;6(2):e18810.

393. Olayiwola JN, Magana C, Harmon A, Nair S, Esposito E, Harsh C, et al. Telehealth as a Bright Spot of the COVID-19 Pandemic: Recommendations From the Virtual Frontlines ("Frontweb"). JMIR Public Health Surveill. 2020;6(2):e19045.

394. Oldenburg R, Marsch A. Optimizing teledermatology visits for dermatology resident education during the COVID-19 pandemic. J Am Acad Dermatol. 2020;82(6):e229.

395. Omboni S. Telemedicine During the COVID-19 in Italy: A Missed Opportunity? Telemed J E Health. 2020;26(8):973-5.

396. Ossami Saidy RR, Globke B, Pratschke J, Schoening W, Eurich D. Successful implementation of preventive measures leads to low relevance of SARS-CoV-2 in liver transplant patients: Observations from a German outpatient department. Transpl Infect Dis. 2020:e13363.

397. Ozdemir V. Special Issue: Digital Health in Times of COVID-19. OMICS. 2020;24(5):229-30.

398. Paleri V, Hardman J, Tikka T, Bradley P, Pracy P, Kerawala C. Rapid implementation of an evidence-based remote triaging system for assessment of suspected referrals and patients with head and neck cancer on follow-up after treatment during the COVID-19 pandemic: Model for international collaboration. Head Neck. 2020;42(7):1674-80.

399. Palomba G, Dinuzzi VP, De Palma GD, Aprea G. Management strategies and role of telemedicine in a surgery unit during COVID-19 outbreak. Int J Surg. 2020;79:189-90.

400. Panzirer D. Role of Non-Profit Organizations During COVID-19 for Diabetes Care: Health Care Inequities and Role of Virtual Specialty Clinic. Diabetes Technol Ther. 2020;22(6):440-3.

401. Pappot N, Taarnhoj GA, Pappot H. Telemedicine and e-Health Solutions for COVID-19: Patients' Perspective. Telemed J E Health. 2020;26(7):847-9.

402. Parikh A, Kumar AA, Jahangir E. Cardio-Oncology Care in the Time of COVID-19 and the Role of Telehealth. JACC CardioOncol. 2020;2(2):356-8.

403. Parisien RL, Shin M, Constant M, Saltzman BM, Li X, Levine WN, et al. Telehealth Utilization in Response to the Novel Coronavirus (COVID-19) Pandemic in Orthopaedic Surgery. J Am Acad Orthop Surg. 2020;28(11):e487-e92.

404. Patel PD, Cobb J, Wright D, Turer RW, Jordan T, Humphrey A, et al. Rapid development of telehealth capabilities within pediatric patient portal infrastructure for COVID-19 care: barriers, solutions, results. J Am Med Inform Assoc. 2020;27(7):1116-20.

405. Patel S, Douglas-Moore J. A reflection on an adapted approach from face-to-face to telephone consultations in our Urology Outpatient Department during the COVID-19 pandemic - a pathway for change to future practice? BJU Int. 2020;126(3):339-41.

406. Peahl AF, Smith RD, Moniz MH. Prenatal care redesign: creating flexible maternity care models through virtual care. Am J Obstet Gynecol. 2020;223(3):389 e1- e10.

407. Peden CJ, Mohan S, Pagan V. Telemedicine and COVID-19: an Observational Study of Rapid Scale Up in a US Academic Medical System. J Gen Intern Med. 2020;35(9):2823-5.

408. Pereira RB. Participating in telehealth is a complex occupation. Aust Occup Ther J. 2020;67(3):284.

409. Perez Sust P, Solans O, Fajardo JC, Medina Peralta M, Rodenas P, Gabalda J, et al. Turning the Crisis Into an Opportunity: Digital Health Strategies Deployed During the COVID-19 Outbreak. JMIR Public Health Surveill. 2020;6(2):e19106.

410. Perez-Alba E, Nuzzolo-Shihadeh L, Espinosa-Mora JE, Camacho-Ortiz A. Use of self-administered surveys through QR code and same center telemedicine in a walk-in clinic in the era of COVID-19. J Am Med Inform Assoc. 2020;27(6):985-6.

411. Perkins S, Cohen JM, Nelson CA, Bunick CG. Teledermatology in the era of COVID-19: Experience of an academic department of dermatology. J Am Acad Dermatol. 2020;83(1):e43-e4.

412. Perniola S, Alivernini S, Varriano V, Paglionico A, Tanti G, Rubortone P, et al. Telemedicine will not keep us apart in COVID-19 pandemic. Ann Rheum Dis. 2020.

413. Perrin PB, Pierce BS, Elliott TR. COVID-19 and telemedicine: A revolution in healthcare delivery is at hand. Health Sci Rep. 2020;3(2):e166.

414. Perrin PB, Rybarczyk BD, Pierce BS, Jones HA, Shaffer C, Islam L. Rapid telepsychology deployment during the COVID-19 pandemic: A special issue commentary and lessons from primary care psychology training. J Clin Psychol. 2020;76(6):1173-85.

415. Perrone G, Zerbo S, Bilotta C, Malta G, Argo A. Telemedicine during Covid-19 pandemic: Advantage or critical issue? Med Leg J. 2020;88(2):76-7.

416. Pollock K, Setzen M, Svider PF. Embracing telemedicine into your otolaryngology practice amid the COVID-19 crisis: An invited commentary. Am J Otolaryngol. 2020;41(3):102490.

417. Portnoy J, Waller M, Elliott T. Telemedicine in the Era of COVID-19. J Allergy Clin Immunol Pract. 2020;8(5):1489-91.

418. Postigo A, Gonzalez-Mansilla A, Bermejo J, Elizaga J, Fernandez-Aviles F, Martinez-Selles M. [Telecardiology in times of the COVID-19 pandemic]. Rev Esp Cardiol. 2020;73(8):674-6.

419. Prasad A, Brewster R, Newman JG, Rajasekaran K. Optimizing your telemedicine visit during the COVID-19 pandemic: Practice guidelines for patients with head and neck cancer. Head Neck. 2020;42(6):1317-21.

420. Prasad A, Carey RM, Rajasekaran K. Head and neck virtual medicine in a pandemic era: Lessons from COVID-19. Head Neck. 2020;42(6):1308-9.

421. Probst T, Stippl P, Pieh C. Changes in Provision of Psychotherapy in the Early Weeks of the COVID-19 Lockdown in Austria. Int J Environ Res Public Health. 2020;17(11).

422. Punia V, Nasr G, Zagorski V, Lawrence G, Fesler J, Nair D, et al. Evidence of a Rapid Shift in Outpatient Practice During the COVID-19 Pandemic Using Telemedicine. Telemed J E Health. 2020;26(10):1301-3.

423. Queen D, Harding K. COVID-19 a short-term challenge, telewound a lifetime change. Int Wound J. 2020;17(3):529.

424. Quek L, Kannivelu A, Pua U. (90)Y Radioembolization: Telemedicine During COVID-19 Outbreak, Opportunity for Prime Time. J Nucl Med. 2020;61(6):780.

425. R P. Will COVID-19 change how we prescribe? Prescriber. 2020;31(5):4.

426. Rabunal R, Suarez-Gil R, Golpe R, Martinez-Garcia M, Gomez-Mendez R, Romay-Lema E, et al. Usefulness of a Telemedicine Tool TELEA in the Management of the COVID-19 Pandemic. Telemed J E Health. 2020.

427. Rajasekaran K. Access to Telemedicine-Are We Doing All That We Can during the COVID-19 Pandemic? Otolaryngol Head Neck Surg. 2020;163(1):104-6.

428. Ramdas K, Ahmed F, Darzi A. Remote shared care delivery: a virtual response to COVID-19. Lancet Digit Health. 2020;2(6):e288-e9.

429. Rametta SC, Fridinger SE, Gonzalez AK, Xian J, Galer PD, Kaufman M, et al. Analyzing 2,589 child neurology telehealth encounters necessitated by the COVID-19 pandemic. Neurology. 2020;95(9):e1257-e66.

430. Ramsetty A, Adams C. Impact of the digital divide in the age of COVID-19. J Am Med Inform Assoc. 2020;27(7):1147-8.

431. Reddy KR. SARS-CoV-2 and the Liver: Considerations in Hepatitis B and Hepatitis C Infections. Clin Liver Dis (Hoboken). 2020;15(5):191-4.

432. Renard E. Personal Experience With COVID-19 and Diabetes in the South of France: Technology Facilitates the Management of Diabetes in Disruptive Times. J Diabetes Sci Technol. 2020;14(4):772-3.

433. Restellini S, Buyse S, Godat S, Goossens N, Maillard MH. [Management of gastrointestinal and hepatic diseases during the COVID-19 outbreak]. Rev Med Suisse. 2020;16(N degrees 691-2):845-8.

434. Rismiller K, Cartron AM, Trinidad JCL. Inpatient teledermatology during the COVID-19 pandemic. J Dermatolog Treat. 2020;31(5):441-3.

435. Robbins T, Hudson S, Ray P, Sankar S, Patel K, Randeva H, et al. COVID-19: A new digital dawn? Digit Health. 2020;6:2055207620920083.

436. Rockwell KL, Gilroy AS. Incorporating telemedicine as part of COVID-19 outbreak response systems. Am J Manag Care.26(4):147-8.

437. Rogers BG, Coats CS, Adams E, Murphy M, Stewart C, Arnold T, et al. Development of Telemedicine Infrastructure at an LGBTQ+ Clinic to Support HIV Prevention and Care in Response to COVID-19, Providence, RI. AIDS Behav. 2020;24(10):2743-7.

438. Romanick-Schmiedl S, Raghu G. Telemedicine - maintaining quality during times of transition. Nat Rev Dis Primers. 2020;6(1):45.

439. Rosen CB, Joffe S, Kelz RR. COVID-19 Moves Medicine into a Virtual Space: A Paradigm Shift From Touch to Talk to Establish Trust. Ann Surg.

440. Roy B, Nowak RJ, Roda R, Khokhar B, Patwa HS, Lloyd T, et al. Teleneurology during the COVID-19 pandemic: A step forward in modernizing medical care. J Neurol Sci. 2020;414:116930.

441. Russo V, Nigro G, D'Onofrio A. COVID-19 and cardiac implantable electronic device remote monitoring: crocodile tears or new opportunity? Expert Rev Med Devices. 2020;17(6):471-2.

442. Saedon H, Gould G, Begum M, Aslam TM. Video Conferencing in the Intravitreal Injection Clinic in Response to the COVID-19 Pandemic. Ophthalmol Ther. 2020;9(3):1-6.

443. Saleem SM, Pasquale LR, Sidoti PA, Tsai JC. Virtual Ophthalmology: Telemedicine in a COVID-19 Era. Am J Ophthalmol. 2020;216:237-42.

444. Saltman DC. Is COVID-19 an opportunity to improve virtual leadership? Aust J Gen Pract. 2020;49.

445. Salzano A, D'Assante R, Stagnaro FM, Valente V, Crisci G, Giardino F, et al. Heart failure management during the COVID-19 outbreak in Italy: a telemedicine experience from a heart failure university tertiary referral centre. Eur J Heart Fail. 2020;22(6):1048-50.

446. Samuels EA, Clark SA, Wunsch C, Jordison Keeler LA, Reddy N, Vanjani R, et al. Innovation During COVID-19: Improving Addiction Treatment Access. J Addict Med. 2020;14(4):e8-e9.

447. Santos-Parker KS, Santos-Parker JR, Highet A, Montgomery JR, Wakam GK, Sonnenday CJ, et al. Practice change amidst the COVID-19 pandemic: Harnessing the momentum for expanding telehealth in transplant. Clin Transplant. 2020:e13897.

448. Santos-Peyret A, Duron RM, Sebastian-Diaz MA, Crail-Melendez D, Gomez-Ventura S, Briceno-Gonzalez E, et al. [E-health tools to overcome the gap in epilepsy care before, during and after COVID-19 pandemics]. Rev Neurol. 2020;70(9):323-8.

449. Sarbadhikari S, Sarbadhikari SN. The global experience of digital health interventions in COVID-19 management. Indian J Public Health. 2020;64(Supplement):S117-S24.

450. Sarti D, De Salvatore M, Gazzola S, Pantaleoni C, Granocchio E. So far so close: an insight into smart working and telehealth reorganization of a Language and Learning Disorders Service in Milan during COVID-19 pandemic. Neurol Sci. 2020;41(7):1659-62.

451. Schwamm LH, Erskine A, Licurse A. A digital embrace to blunt the curve of COVID19 pandemic. NPJ Digit Med. 2020;3(1):64.

452. Scott BK, Miller GT, Fonda SJ, Yeaw RE, Gaudaen JC, Pavliscsak HH, et al. Advanced Digital Health Technologies for COVID-19 and Future Emergencies. Telemed J E Health. 2020;26(10):1226-33.

453. Seah KM. COVID-19: Exposing digital poverty in a pandemic. Int J Surg. 2020;79:127-8.

454. Seifert A. The Digital Exclusion of Older Adults during the COVID-19 Pandemic. J Gerontol Soc Work. 2020:1-3.

455. Sell E, Chao T, Shah M, Rajasekaran K. Creation of Educational Videos for Patients Undergoing Nonelective Surgery: Tools for the COVID-19 Era. Otolaryngol Head Neck Surg. 2020;163(1):83-5.

456. Sell NM, Silver JK, Rando S, Draviam AC, Mina DS, Qadan M. Prehabilitation Telemedicine in Neoadjuvant Surgical Oncology Patients During the Novel COVID-19 Coronavirus Pandemic. Ann Surg. 2020;272(2):e81-e3.

457. Serper M, Cubell AW, Deleener ME, Casher TK, Rosenberg DJ, Whitebloom D, et al. Telemedicine in Liver Disease and Beyond: Can the COVID-19 Crisis Lead to Action? Hepatology.

458. Setzen M, Svider PF, Pollock K. COVID-19 and rhinology: A look at the future. Am J Otolaryngol. 2020;41(3):102491.

459. Shah M, Sachdeva M, Alavi A, Shi VY, Hsiao JL. Optimizing care for atopic dermatitis patients during the COVID-19 pandemic. J Am Acad Dermatol. 2020;83(2):e165-e7.

460. Shalev D, Shapiro PA. Epidemic psychiatry: The opportunities and challenges of COVID-19. Gen Hosp Psychiatry. 2020;64:68-71.

461. Sharma A, Jindal V, Singla P, Goldust M, Mhatre M. Will teledermatology be the silver lining during and after COVID-19? Dermatol Ther. 2020:e13643.

462. Shenoy P, Ahmed S, Paul A, Skaria TG, Joby J, Alias B. Switching to teleconsultation for rheumatology in the wake of the COVID-19 pandemic: feasibility and patient response in India. Clin Rheumatol. 2020;39(9):2757-62.

463. Shew A. Let COVID-19 expand awareness of disability tech. Nature. 2020;581(7806):9.

464. Shipchandler TZ, Nesemeier BR, Parker NP, Vernon D, Campiti VJ, Anthony BP, et al. Telehealth Opportunities for the Otolaryngologist: A Silver Lining During the COVID-19 Pandemic. Otolaryngol Head Neck Surg. 2020;163(1):112-3.

465. Shirke MM, Shaikh SA, Harky A. Tele-oncology in the COVID-19 Era: The Way Forward? Trends Cancer. 2020;6(7):547-9.

466. Shokri T, Lighthall JG. Telemedicine in the Era of the COVID-19 Pandemic: Implications in Facial Plastic Surgery. Facial Plast Surg Aesthet Med. 2020;22(3):155-6.

467. Shura RD, Brearly TW, Tupler LA. Telehealth in Response to the COVID-19 Pandemic in Rural Veteran and Military Beneficiaries. J Rural Health. 2020.

468. Simpson CL, Kovarik CL. Effectively Engaging Geriatric Patients via Teledermatology. J Am Acad Dermatol. 2020.

469. Singh RP, Javaid M, Kataria R, Tyagi M, Haleem A, Suman R. Significant applications of virtual reality for COVID-19 pandemic. Diabetes Metab Syndr. 2020;14(4):661-4.

470. Skayem C, Cassius C, Ben Kahla M, Fiani C, Frumholtz L, Mrad M, et al. Teledermatology for COVID-19 cutaneous lesions: substitute or supplement? J Eur Acad Dermatol Venereol. 2020;34(10):e532-e3.

471. Smith AC, Thomas E, Snoswell CL, Haydon H, Mehrotra A, Clemensen J, et al. Telehealth for global emergencies: Implications for coronavirus disease 2019 (COVID-19). J Telemed Telecare. 2020;26(5):309-13.

472. Smith WR, Atala AJ, Terlecki RP, Kelly EE, Matthews CA. Implementation Guide for Rapid Integration of an Outpatient Telemedicine Program During the COVID-19 Pandemic. J Am Coll Surg. 2020;231(2):216-22 e2.

473. Sodhi M. Telehealth Policies Impacting Federally Qualified Health Centers in Face of COVID-19. J Rural Health. 2020.

474. Song X, Liu X, Wang C. The role of telemedicine during the COVID-19 epidemic in China-experience from Shandong province. Crit Care. 2020;24(1):178.

475. Sousa A, Karia S. Telepsychiatry during COVID-19: Some clinical, public health, and ethical dilemmas. Indian J Public Health. 2020;64(Supplement):S245-S6.

476. Srinivasan SR. Editorial: Tele-ICU in the Age of COVID-19: Built for This Challenge. J Nutr Health Aging. 2020;24(5):536-7.

477. Sterpetti AV. COVID-19 diffusion capability is its worst, unpredictable chracateristic. How to visit a patient from a distance. Br J Surg. 2020;107(7):e181.

478. Stoessl AJ, Bhatia KP, Merello M. Movement Disorders in the World of COVID-19. Mov Disord. 2020;35(5):709-10.

479. Sun S, Yu K, Xie Z, Pan X. China empowers Internet hospital to fight against COVID-19. J Infect. 2020;81(1):e67-e8.

480. Sutherland AE, Stickland J, Wee B. Can video consultations replace face-to-face interviews? Palliative medicine and the Covid-19 pandemic: rapid review. BMJ Support Palliat Care. 2020;10(3):271-5.

481. Swierad M, Dyrbus K, Szkodzinski J, Zembala MO, Kalarus Z, Gasior M. Telehealth visits in a tertiary cardiovascular center as a response of the healthcare system to the severe acute respiratory syndrome coronavirus 2 pandemic in Poland. Pol Arch Intern Med. 2020;130(7-8):700-3.

482. Szmuda T, Ali S, Sloniewski P, Group NW. Telemedicine in neurosurgery during the novel coronavirus (COVID-19) pandemic. Neurol Neurochir Pol. 2020;54(2):207-8.

483. Szperka CL, Ailani J, Barmherzig R, Klein BC, Minen MT, Halker Singh RB, et al. Migraine Care in the Era of COVID-19: Clinical Pearls and Plea to Insurers. Headache. 2020;60(5):833-42.

484. Tack CCJ. Would You Believe? A Virus Changes Diabetes Care. J Diabetes Sci Technol. 2020;14(4):795-6.

485. Tanaka MJ, Oh LS, Martin SD, Berkson EM. Telemedicine in the Era of COVID-19: The Virtual Orthopaedic Examination. J Bone Joint Surg Am. 2020;102(12):e57.

486. Tanguturi VK, Lindman BR, Pibarot P, Passeri JJ, Kapadia S, Mack MJ, et al. Managing Severe Aortic Stenosis in the COVID-19 Era. JACC Cardiovasc Interv. 2020;13(16):1937-44.

487. Tashkandi E, Zeeneldin A, AlAbdulwahab A, Elemam O, Elsamany S, Jastaniah W, et al. Virtual Management of Patients With Cancer During the COVID-19 Pandemic: Web-Based Questionnaire Study. J Med Internet Res. 2020;22(6):e19691.

488. Tay YH, Lim L, Cheng A, Sim K. Disrupting the disruption: Using digital tools to support psychiatry residency training in Singapore during the COVID-19 pandemic. Psychiatry Res. 2020;289:113063.

489. Taylor CB, Fitzsimmons-Craft EE, Graham AK. Digital technology can revolutionize mental health services delivery: The COVID-19 crisis as a catalyst for change. Int J Eat Disord. 2020;53(7):1155-7.

490. Taylor PC. Adopting PROs in virtual and outpatient management of RA. Nat Rev Rheumatol. 2020;16(9):477-8.

491. Teles M, Sacchetta T, Matsumoto Y. COVID-19 Pandemic Triggers Telemedicine Regulation and Intensifies Diabetes Management Technology Adoption in Brazil. J Diabetes Sci Technol. 2020;14(4):797-8.

492. Tenderich A. Virtual Nation: Telemedicine's Breakout Moment. J Diabetes Sci Technol. 2020;14(4):799-800.

493. Tenforde AS, Iaccarino MA, Borgstrom H, Hefner JE, Silver J, Ahmed M, et al. Telemedicine During COVID-19 for Outpatient Sports and Musculoskeletal Medicine Physicians. PM R. 2020;12(9):926-32.

494. Thomas E, Gallagher R, Grace SL. Future-proofing cardiac rehabilitation: Transitioning services to telehealth during COVID-19. Eur J Prev Cardiol. 2020:2047487320922926.

495. Thornton J. The "virtual wards" supporting patients with covid-19 in the community. BMJ. 2020;369:m2119.

496. Thota R, Gill DM, Brant JL, Yeatman TJ, Haslem DS. Telehealth Is a Sustainable Population Health Strategy to Lower Costs and Increase Quality of Health Care in Rural Utah. JCO Oncol Pract. 2020;16(7):e557-e62.

497. Thulesius H. Increased importance of digital medicine and eHealth during the Covid-19 pandemic. Scand J Prim Health Care. 2020;38(2):105-6.

498. Ting DSW, Carin L, Dzau V, Wong TY. Digital technology and COVID-19. Nat Med. 2020;26(4):459-61.

499. Toft AD. Learning From COVID-19 and Looking to Technology Ahead. J Diabetes Sci Technol. 2020;14(4):801-2.

500. Tolone S, Gambardella C, Brusciano L, Del Genio G, Lucido FS, Docimo L. Telephonic triage before surgical ward admission and telemedicine during COVID-19 outbreak in Italy. Effective and easy procedures to reduce in-hospital positivity. Int J Surg. 2020;78:123-5.

501. Torous J, Jan Myrick K, Rauseo-Ricupero N, Firth J. Digital Mental Health and COVID-19: Using Technology Today to Accelerate the Curve on Access and Quality Tomorrow. JMIR Ment Health. 2020;7(3):e18848.

502. Trethewey SP, Beck KJ, Symonds RF. Video consultations in UK primary care in response to the COVID-19 pandemic. Br J Gen Pract. 2020;70(694):228-9.

503. Triana AJ, Gusdorf RE, Shah KP, Horst SN. Technology Literacy as a Barrier to Telehealth During COVID-19. Telemed J E Health. 2020;26(9):1118-9.

504. Triantafillou V, Rajasekaran K. A Commentary on the Challenges of Telemedicine for Head and Neck Oncologic Patients during COVID-19. Otolaryngol Head Neck Surg. 2020;163(1):81-2.

505. Trinidad J, Kroshinsky D, Kaffenberger BH, Rojek NW. Telemedicine for inpatient dermatology consultations in response to the COVID-19 pandemic. J Am Acad Dermatol. 2020;83(1):e69-e71.

506. Tullio V, Perrone G, Bilotta C, Lanzarone A, Argo A. Psychological support and psychotherapy via digital devices in Covid-19 emergency time: Some critical issues. Med Leg J. 2020;88(2):73-6.

507. Turer RW, Jones I, Rosenbloom ST, Slovis C, Ward MJ. Electronic personal protective equipment: A strategy to protect emergency department providers in the age of COVID-19. J Am Med Inform Assoc. 2020;27(6):967-71.

508. Turolla A, Rossettini G, Viceconti A, Palese A, Geri T. Musculoskeletal Physical Therapy During the COVID-19 Pandemic: Is Telerehabilitation the Answer? Phys Ther. 2020;100(8):1260-4.

509. Unutzer J, Kimmel RJ, Snowden M. Psychiatry in the age of COVID-19. World Psychiatry. 2020;19(2):130-1.

510. Vaira LA, Salzano G, Petrocelli M, Deiana G, Salzano FA, De Riu G. Validation of a self-administered olfactory and gustatory test for the remotely evaluation of COVID-19 patients in home quarantine. Head Neck. 2020;42(7):1570-6.

511. Valentino LA, Skinner MW, Pipe SW. The role of telemedicine in the delivery of health care in the COVID-19 pandemic. Haemophilia. 2020.

512. Verstraete SG, Sola AM, Ali SA. Telemedicine for Pediatric Inflammatory Bowel Disease in the Era of COVID-19. J Pediatr Gastroenterol Nutr. 2020;70(6):e140.

513. Vidal-Alaball J, Acosta-Roja R, Pastor Hernandez N, Sanchez Luque U, Morrison D, Narejos Perez S, et al. Telemedicine in the face of the COVID-19 pandemic. Aten Primaria. 2020;52(6):418-22.

514. Vigano M, Voza A, Harari S, Eusebio A, Ripoll Pons M, Bordonali M, et al. Letter to the Editor: Clinical Management of Nonrespiratory Diseases in the COVID-19 Pandemic: What Have We Done and What Needs to Be Done? Telemed J E Health. 2020;26(10):1206-8.

515. Vilendrer S, Patel B, Chadwick W, Hwa M, Asch S, Pageler N, et al. Rapid Deployment of Inpatient Telemedicine In Response to COVID-19 Across Three Health Systems. J Am Med Inform Assoc. 2020;27(7):1102-9.

516. Villa A, Sankar V, Shiboski C. Tele(oral)medicine: A new approach during the COVID-19 crisis. Oral Dis. 2020.

517. Villani A, Annunziata MC, Abategiovanni L, Fabbrocini G. Teledermatology for acne patients: How to reduce face-to-face visits during COVID-19 pandemic. J Cosmet Dermatol. 2020;19(8):1828.

518. Villani A, Scalvenzi M, Fabbrocini G. Teledermatology: a useful tool to fight COVID-19. J Dermatolog Treat. 2020;31(4):325.

519. Visca D, Tiberi S, Pontali E, Spanevello A, Migliori GB. Tuberculosis in the time of COVID-19: quality of life and digital innovation. Eur Respir J. 2020;56(2).

520. Vokinger KN, Nittas V, Witt CM, Fabrikant SI, von Wyl V. Digital health and the COVID-19 epidemic: an assessment framework for apps from an epidemiological and legal perspective. Swiss Med Wkly. 2020;150:w20282.

521. Waki K, Sankoda A, Amano S, Ogawa M, Ohe K. Responding to COVID-19: Agile Use of Information Technology to Serve Patients With Diabetes. J Diabetes Sci Technol. 2020;14(4):807-8.

522. Waller G, Pugh M, Mulkens S, Moore E, Mountford VA, Carter J, et al. Cognitive-behavioral therapy in the time of coronavirus: Clinician tips for working with eating disorders via telehealth when face-to-face meetings are not possible. Int J Eat Disord. 2020;53(7):1132-41.

523. Wang SS, Roubidoux MA. Coronavirus Disease 2019 (COVID-19), Videoconferencing, and Gender. J Am Coll Radiol. 2020;17(7):918-20.

524. Wang SSY, Teo WZW, Teo WZY, Chai YW. Virtual Reality as a Bridge in Palliative Care during COVID-19. J Palliat Med. 2020;23(6):756.

525. Watson AR, Wah R, Thamman R. The Value of Remote Monitoring for the COVID-19 Pandemic. Telemed J E Health. 2020;26(9):1110-2.

526. Webster P. Virtual health care in the era of COVID-19. Lancet. 2020;395(10231):1180-1.

527. Whaibeh E, Mahmoud H, Naal H. Telemental Health in the Context of a Pandemic: the COVID-19 Experience. Curr Treat Options Psychiatry. 2020:1-5.

528. Whelan P, Stockton-Powdrell C, Jardine J, Sainsbury J. Comment on "Digital Mental Health and COVID-19: Using Technology Today to Accelerate the Curve on Access and Quality Tomorrow": A UK Perspective. JMIR Ment Health. 2020;7(4):e19547.

529. Wind TR, Rijkeboer M, Andersson G, Riper H. The COVID-19 pandemic: The 'black swan' for mental health care and a turning point for e-health. Internet Interventions. 2020;20:100317.

530. Wittbold KA, Baugh JJ, Yun BJ, Raja AS, White BA. iPad deployment for virtual evaluation in the emergency department during the COVID-19 pandemic. Am J Emerg Med. 2020.

531. Wong JKW, Shih KC, Chan JCH, Lai JSM. Tele-ophthalmology amid COVID-19 pandemic-Hong Kong experience. Graefes Arch Clin Exp Ophthalmol. 2020:1.

532. Woo Baidal JA, Chang J, Hulse E, Turetsky R, Parkinson K, Rausch JC. Zooming Toward a Telehealth Solution for Vulnerable Children with Obesity During Coronavirus Disease 2019. Obesity (Silver Spring). 2020;28(7):1184-6.

533. Woolliscroft JO. Innovation in Response to the COVID-19 Pandemic Crisis. Acad Med. 2020;95(8):1140-2.

534. Wosik J, Fudim M, Cameron B, Gellad ZF, Cho A, Phinney D, et al. Telehealth transformation: COVID-19 and the rise of virtual care. J Am Med Inform Assoc. 2020;27(6):957-62.

535. Wright JH, Caudill R. Remote Treatment Delivery in Response to the COVID-19 Pandemic. Psychother Psychosom. 2020;89(3):130-2.

536. Xie B, Charness N, Fingerman K, Kaye J, Kim MT, Khurshid A. When Going Digital Becomes a Necessity: Ensuring Older Adults' Needs for Information, Services, and Social Inclusion During COVID-19. J Aging Soc Policy. 2020;32(4-5):460-70.

537. Yellowlees P, Nakagawa K, Pakyurek M, Hanson A, Elder J, Kales HC. Rapid Conversion of an Outpatient Psychiatric Clinic to a 100% Virtual Telepsychiatry Clinic in Response to COVID-19. Psychiatr Serv. 2020;71(7):749-52.

538. Yemm KE, Arnall JR, Cowgill NA. Necessity of pharmacist-driven nonprescription telehealth consult services in the era of COVID-19. Am J Health Syst Pharm. 2020;77(15):1188.

539. Young SD, Schneider J. Clinical Care, Research, and Telehealth Services in the Era of Social Distancing to Mitigate COVID-19. AIDS Behav. 2020;24(7):2000-2.

540. Zhai Y. A Call for Addressing Barriers to Telemedicine: Health Disparities during the COVID-19 Pandemic. Psychother Psychosom. 2020:1-3.

541. Zhou X, Snoswell CL, Harding LE, Bambling M, Edirippulige S, Bai X, et al. The Role of Telehealth in Reducing the Mental Health Burden from COVID-19. Telemed J E Health. 2020;26(4):377-9.

542. Zubatsky M, Berg-Weger M, Morley J. Using Telehealth Groups to Combat Loneliness in Older Adults Through COVID-19. J Am Geriatr Soc. 2020;68(8):1678-9.

543. Zughni LA, Gillespie AI, Hatcher JL, Rubin AD, Giliberto JP. Telemedicine and the Interdisciplinary Clinic Model: During the COVID-19 Pandemic and Beyond. Otolaryngol Head Neck Surg. 2020;163(4):673-5.
