# Supplementary material for: Use of Telehealth During the COVID-19 Pandemic: Scoping Review
Source: J Med Internet Res. 2020 Dec 1;22(12):e24087. doi: 10.2196/24087 (PMC7710390; doi:10.2196/24087)
Supplement: Multimedia Appendix 4 [file jmir_v22i12e24087_app4.pdf]

Multimedia appendix 4

Table S1
World Health Organization Classification

Africa	Number (Frequency %)
N/A	N/A
**Total**	**0 (0)**
South East Asia	Number (Frequency %)
India	25 (4.6)
Bangladesh	1 (0.2)
N/A	N/A
**Total**	**26**
Eastern Mediterranean	Number (Frequency %)
Iran	8 (1.5)
Pakistan	4 (0.7)
Lebanon	2 (0.4)
Afghanistan	1 (0.2)
Jordan	1 (0.2)
UAE	1 (0.2)
Saudi Arabia	1 (0.2)
**Total**	**18 (3.4)**

Americas	Number (Frequency %)
USA	237 (43.6)
Canada	25 (4.6)
Brazil	10 (1.8)
Mexico	3 (0.6)
Chile	1 (0.2)
Argentina	1 (0.2)
**Total**	**277 (51)**

Europe	Number (Frequency %)
UK	52
Italy	44
France	15
Spain	13
Switzerland	7
Germany	6
Ireland	5
Greece	4

Denmark	4
Netherlands	3
Austria	2
Norway	2
Poland	2
Sweden	2
Turkey	2
Belgium	1
Croatia	1
Hungary	1
Luxembourg	1
Portugal	1
**Total**	**168**

Western Pacific	Number (Frequency %)
Australia	16 (2.9)
China	15 (2.8)
Singapore	11 (2)
Japan	5 (0.9)
South Korea	4 (0.7)
New Zealand	2 (0.4)
Taiwan	1 (0.2)
**Total**	**54 (9.9)**

Table S2
World Bank Classification

Low income countries	Number (Frequency %)
Afghanistan	1 (0.2)
N/A	N/A
**Total**	**1 (0.2)**
Lower-middle income countries	Number (Frequency %)
India	25 (4.6)
Pakistan	4 (0.7)
Bangladesh	1 (0.2)
**Total**	**30 (5.5)**
Upper-middle income countries	Number (Frequency %)
China	15 (2.8)
Brazil	10 (1.8)
Iran	8 (1.5)
Mexico	3 (0.6)
Lebanon	2 (0.4)
Turkey	2 (0.4)
Argentina	1 (0.2)
Jordan	1 (0.2)
**Total**	**42 (7.9)**
High income countries	Number (Frequency %)
USA	237 (43.6)
UK	52 (9.5)
Italy	44 (8.1)
Canada	25 (4.6)
Australia	16 (2.9)
France	15 (2.8)
Spain	13 (2.4)
Singapore	11 (2)
Switzerland	7 (1.3)
Germany	6 (1.1)
Ireland	5 (0.9)
Japan	5 (0.9)
Greece	4 (0.7)
Denmark	4 (0.7)
South Korea	4 (0.7)
Netherlands	3 (0.6)
Austria	2 (0.4)
New Zealand	2 (0.4)
Norway	2 (0.4)
Poland	2 (0.4)

Sweden	2 (0.4)
UAE	1 (0.2)
Belgium	1 (0.2)
Chile	1 (0.2)
Croatia	1 (0.2)
Hungary	1 (0.2)
Luxembourg	1 (0.2)
Portugal	1 (0.2)
Saudi Arabia	1 (0.2)
Taiwan	1 (0.2)
**Total**	**470 (86.6)**

Table S3
World Health Organization Classification

Africa	Number (Frequency %)
N/A	N/A
**Total**	**0 (0)**
South East Asia	Number (Frequency %)
India	19 (3.5)
Bangladesh	1 (0.2)
N/A	N/A
**Total**	**20**

Eastern Mediterranean	Number (Frequency %)
Iran	6 (1)
Pakistan	4 (0.7)
Lebanon	1 (0.2)
Jordan	1 (0.2)
Afghanistan	1 (0.2)
**Total**	**13 (2.3)**

Americas	Number (Frequency %)
USA	178 (33)
Canada	18 (3.2)
Brazil	9 (1.7)
Chile	1 (0.2)
Peru	1 (0.2)
Mexico	1 (0.2)
Argentina	1 (0.2)
**Total**	**210 (38.7)**

Europe	Number (Frequency %)
UK	33 (6)
Italy	31 (5.7)
France	12 (2.1)
Spain	9 (1.7)
Germany	6 (1.1)
Switzerland	4 (0.7)
Greece	2 (0.4)
Denmark	2 (0.4)
Norway	2 (0.4)
Austria	1 (0.2)
Croatia	1 (0.2)
Hungary	1 (0.2)

Luxembourg	1 (0.2)
Netherlands	1 (0.2)
Portugal	1 (0.2)
Sweden	1 (0.2)
Turkey	1 (0.2)
**Total**	**108**

Western Pacific	Number (Frequency %)
Australia	11 (1.9)
China	11 (1.9)
Singapore	5 (0.9)
Japan	4 (0.7)
South Korea	3 (0.6)
New Zealand	2 (0.4)
Taiwan	1 (0.2)
**Total**	**37 (6.7)**

Global / Multicountry	Number (Frequency %)
	155 (28.6)
**Total**	**155 (28.6)**

Table S4
World Bank Classification

Low income countries	Number (Frequency %)
Afghanistan	1 (0.2)
N/A	N/A
**Total**	**1 (0.2)**

Lower-middle income countries	Number (Frequency %)
India	19 (3.5)
Pakistan	4 (0.7)
Bangladesh	1 (0.2)
**Total**	**24 (4.4)**

Upper-middle income countries	Number (Frequency %)
China	11 (2)
Brazil	9 (1.7)
Iran	6 (1)
Argentina	1 (0.2)
Jordan	1 (0.2)
Lebanon	1 (0.2)
Mexico	1 (0.2)
Peru	1 (0.2)
Turkey	1 (0.2)
**Total**	**32 (5.9)**

High income countries	Number (Frequency %)
USA	178 (32.8)
UK	33 (6)
Italy	31 (5.7)
Canada	18 (3.2)
France	12 (2.1)
Australia	11 (2)
Spain	9 (1.7)
Germany	6 (1.1)
Singapore	5 (0.7)
Switzerland	4 (0.7)
Japan	4 (0.7)
South Korea	3 (0.6)
Greece	2 (0.4)
Denmark	2 (0.4)
New Zealand	2 (0.4)

Norway	2 (0.4)
Austria	1 (0.2)
Chile	1 (0.2)
Croatia	1 (0.2)
Hungary	1 (0.2)
Luxembourg	1 (0.2)
Netherlands	1 (0.2)
Portugal	1 (0.2)
Sweden	1 (0.2)
Taiwan	1 (0.2)
**Total**	**331 (60.9)**
