# Supplementary material for: Use of Telehealth During the COVID-19 Pandemic: Scoping Review
Source: J Med Internet Res. 2020 Dec 1;22(12):e24087. doi: 10.2196/24087 (PMC7710390; doi:10.2196/24087)
Supplement: Multimedia Appendix 5 [file jmir_v22i12e24087_app5.doc]

**Multimedia appendix 5**

‘‘health web science’’— a combination of telemedicine and e-health

…there are three primary forms of telehealth, including video,

“the delivery of health care services, where distance is a critical factor, by all health care professionals using information and communication technologies....”

"Digital health: three essential ingredients are noteworthy: (1) ubiquitous planetary digital connectivity enabled

"e-visits: a form of asynchronous telemedicine that could be obtained on the portal

"Provider and patient in immediate proximity; this model is referred to as face-to face care; non–face-to-face e/m care; medical services can be apportioned as procedural care (eg, surgery, radiology, or laboratory testing and others) or cognitive care, also known as evaluation and management (e/m) services, in which the provider formulates an assessment and plan after obtaining information

"Proximity tracing apps: technology to store close contacts over a certain period; may include an automated warning system of contacts in case of covid-19 diagnosis

"Standard telemedicine (with video conferencing), telehealth (with remote monitoring of clinical measurements), telecoaching, and telecare (technology to support independent living)"

"Telehealth (virtual visits, virtual care), mobile apps (remote patient monitoring), and websites and chatbots (risk assessment, screening, triage)"

"Telehealth encompasses a broad range of electronic information and telecommunication technologies to support long-distance clinical health care and related activities through videoconferencing, internet-based applications, store-and-forward imaging, streaming media, and

"Telehealth, it affirms, is “the means by which technologies and related services concerned with health and well-being are accessed by people

"Telehealth: the use of telecommunications and virtual technology to deliver health care outside of traditional health care facilities’

"Tele hospice, which is the delivery of hospice care using remote communication tools.2"

"Telemedicine = remote evaluation and monitoring to diagnose and treat patients"

"Telemedicine = traditional physician-patient (and physician-physician) interactions, enhanced by two-way video and audio communications; the use of information and communications technologies (icts) was extended to support services, training, and health information activities for multidisciplinary healthcare providers and patients, shaping an expanded field called “telehealth”"

"Telemedicine as the use of electronic and telecommunications technologies to provide and support healthcare when distance separates the participants telehealth visits: a visit between a provider and new or established patient using audio and visual telecommunications.

"Telemedicine is the use of telecommunications to diagnose and treat diseases"

"Telemedicine refers to the use of technology such as video conferencing to deliver health

"Telemedicine utilizes information and telecommunications technology to transfer medical information for diagnosis, therapy, and education. "

"Telemedicine, which is also used synonymously with “remote

"Telemedicine: a variety of technologies to securely deliver remote health care

"Telemedicine: this virtual platform could be used by smartphones or webcam-enabled computers and allows physicians to effectively screen patients with early signs of covid-19 before they reach to hospital.

"Telerehabilitation refers to “providing rehabilitation service using electronic

"The definition of telehealth offered here is encompassed by the term digital health.

"The global observatory of ehealth has defined mobile health (mhealth) as medical or public health practice supported by mobile devices

"The literal meaning of the term “telemedicine” is “healing at a distance”. who has defined it as “the delivery of health care services, where distance is a critical factor, by all health care professionals using information and communication technologies for the exchange of valid information for diagnosis, treatment and prevention of disease and injuries, research and evaluation, and for the continuing education of health care providers, all in the interests of advancing the health of individuals and their communities”"

"The traditional practice of side-by-side attending-trainee review of diagnostic imaging exams and case-based teaching done remotely

"Video visits are a real-time, simultaneous audio and visual interaction with patients via a video conferencing platform; an econsult, or interprofessional consultation, is defined as a diagnosis or management service provided by a consulting physician to a requesting physician or other qualified healthcare professional via verbal and written report"

"Video visits: a live simultaneous audio–visual visit between patient and provider using teleconferencing software.

"Virtual care has been defined as any interaction occurring remotely between patients and/or members of their circle of care, through any form of communication or information technology with the aim of facilitating or maximizing the quality and effectiveness of patient care

“the means by which technologies and related services concerned with health and well-being are accessed by people or provided for them irrespective of location.” this definition fits with wootton’s description of telemedicine as “health care carried out at a distance,” with both reflecting a person- (or patient-) centered, as opposed to a technology-driven, approach. "

(iot), (2) sensors embedded in pretty much anything you can imagine, for example, smart watches that monitor heart rate and rhythm and thus, creating and communicating big data, (3) technologies such as artificial intelligence (ai) that allow real-time data analysis and sense making from the big data on human biology as well as ecological systems in which humans are situated. "

Artificial intelligence & text processing: ai used to make predictions of covid-19 tests based on reported symptoms and identify cases

Audio-only visits: patient portals and messaging technologies"

By new tools and concepts such as the internet of things care remotely, without an in-person visit.

Crowd-sourced surveillance: platform to monitor symptom occurrence on population level

Delivery of health care services at a distance using electronic means for “the diagnosis of, treatment, and prevention of disease and injuries, research and evaluation, education of health care providers”

Digital health = technology that, “connects and empowers people and populations to manage health and wellness, augmented by accessible and supportive provider teams working within flexible, integrated, interoperable, and digitally-enabled care environments that strategically leverage digital tools, technologies, and services to transform care delivery”

Digital health is broadly defined as “the field of knowledge and practice associated with the development and use of digital technologies to improve health” [3] across the full range of health technologies introduced into care, including telehealth, mobile health apps and wearable technologies, and online health services and tools.

Digital health: the field of knowledge and practice, associated with any aspect of adopting digital technologies to improve healthcare, from inception to operation

Digital pathology: pathologists view the scanned whole slide images (wsi), some of which are based on scanners that have already been approved by regulatory agencies for routine reporting

Digital physical therapist practice as health care services, support, and information provided remotely via digital communication and devices

Digital practice as “a term used to describe health care services, support, and information provided remotely via digital communication and devices”; “to facilitate effective delivery of physical therapy services by improving access to care and information and managing health care resources.”

e-consults: a mechanism to electronically obtain specialty input clinician- to-clinician"

e-visits: communication initiated by an established patient with the provider through an online patient portal"

e‐scales transfer weight data directly to research/clinical centers through the cellular network, wireless internet, or pairing with a blue tooth device that has internet access.

econsult (interprofessional consult): a written electronic communication between referring provider and consulting physician involving review of a patient’s medical record and treatment recommendations."

electronic communication technologies rather than through in person meetings between a patient and a doctor.4"

Electronic intensive care unit (e-icu) monitoring programs, which allow nurses and physicians to remotely monitor the status of 60 to 100 patients in multiple hospitals

ePPE: telemedicine tools used by on-site emergency providers to evaluate patients physically in the ed to avoid physical proximity.

evisit: a communication between patient and provider via an online patient portal.

Forward triage is the process of determining the patients’ condition before they arrive in the emergency department"

Forward triage: the sorting of patients before they arrive in the emergency department (ed); community paramedicine or mobile integrated health care programs: patients to be treated in their homes, with higher-level medical support provided virtually.

Live (synchronous) videoconferencing: typically, a two-way audio-visual link between a patient and an hcp in different locations

medical care,” refers to providing clinical healthcare through

Mental health interventions: technologies for behavioural interventions, alleviating fears, improvement of mental health"

mhealth or digital health is usually defined as the practice of medicine, public health, and clinical health psychology supported by mobile communication devices, such as mobile phones, tablet computers, wearable activity trackers, and other tools for health services and information.; a subset of digital health often called electronic health

(ehealth) includes health information technology, telemedicine, and personalized medicine or provided for them irrespective of location.” this definition fits with Wootton’s description of telemedicine as “health care carried out at a distance,” with both reflecting a person- (or patient-) cantered, as opposed to a technology-driven, approach. "

Patient diaries: keeping track of symptoms and exposure risks (sometimes combined with population-based symptom surveillance)

Personal videoconferencing, also referred to as e-visit, is the use of personal internet-enabled devices like smartphones and tablets to videoconference with patients, with the goal to keep the patient at home or their preferred location."

Remote learning through broadcast sign-outs, didactics, and digital teaching slides

Remote or non–face-to-face care models, in which the patient is geographically separated from the physician or other qualified health care professional, are enabled by communication-based technologies. “telehealth” or “telemedicine,” from the Greek word for “far.”; many think of telemedicine as consisting exclusively of real-time interactive or synchronous audio and video communications between a patient and a provider. there is a wide spectrum of other telehealth services including telephone communication; asynchronous digital services including email, text messages, and remote monitoring of patient data such as images or physiologic parameters; and provider-to-provider communications

Smartphone-enabled wireless otoscope-assisted online telemedicine (sewoaot): device smartphone-enabled otoscope (seo) with a dedicated smartphone app

Use of mobile phones, patient monitoring devices, pdas, and other wireless devices

Synchronous audio/video interactions, also known as virtual visits (vvs)

synchronous: attending and trainee participate in real-time review of images and discussion of preliminary report utilizing videoconferencing / screensharing technology asynchronous: attending reviews images and preliminary report separately from trainee, edits and finalizes report and provides trainee feedback at a later time"

Tele genetics: common approaches to providing genetic services via telehealth, sometimes referred to as tele genetics, include counseling via telephone or video

Tele mental health: use of information and communications technologies, including videoconferencing, to deliver mental health care remotely, including evaluations, medication management, and psychotherapy

Tele oncology: the application of telemedicine to oncology; virtual prescription and delivery of drugs

Tele palliative medicine as the remote delivery of palliative care services has rapidly expanded in recent years largely in outpatient and home-based settings, with the highest increase in video-based services

Tele-health: advisory platforms and self-assessments in case of symptoms, online consultation with medical experts

Teleconsultation as a “remote medical consultation, mediated by technologies, with doctor and patient located in different geographical spaces

Teleconsultation refers to the electronic communication between a physician and a patient or between two physicians for the purpose of diagnosis and/or treatment

Telehealth encounter as using telecommunication technology to engage in live synchronous audio and videoconferencing using an internet-based connection from any location as part of our institution’s Jeff connect program.

Telehealth is different from telemedicine because it refers to a broader scope of remote healthcare services than telemedicine. while telemedicine refers specifically to remote clinical services, telehealth can refer to remote non-clinical services, such as provider training, administrative meetings, and continuing medical education, in addition to clinical services.

Telehealth system (i.e. video conference software with high resolution cameras)

Telehealth uses digital and telecommunications tools—including telephone, secure messages, and video technology—to manage patient care, health-related education, public health, and health administration

Telehealth: assessment allowing patients to safely remain within the confines of their own home or care facility.

Telehealth: the entire spectrum of activities used to deliver care at a distance—without direct physical contact with the patient. telehealth encompasses both provider-to-patient and provider-to-provider communications, and can take place synchronously (telephone and video), asynchronously (patient portal messages, e-consults), and through virtual agents (chatbots) and wearable devices

Telehealth: the use of messaging, audio, and video communication platforms

Telehealth: treatment that is delivered either by audio and/or video call

Telehealth: web-based patient record system (my health evet) and over 3 dozen mobile apps addressing a wide variety of medical conditions

Telemedicine = tool for delivery of health care services at distance

Telemedicine = videoconference

Telemedicine allows the provision of healthcare remotely using electronic communication tools. virtual clinic is a form of telemedicine between healthcare professionals and patients that crucially occurs without the need for a traditional face‐to‐face consultation and thus avoids in‐person attendance at hospital

Telemedicine can be defined as medical care provided to a patient by a physician or other health care provider at a different location; real-time interactive audio and video telecommunication systems.

Telemedicine in neurology - Teleneurology

Telemedicine is defined as ‘the use of telecommunication technology to provide medical information and services.

Telemedicine is defined as the remote diagnosis and treatment of patients through telecommunications technology

Telemedicine is defined as the use of electronic information and communication technologies to provide healthcare support when distance separates patients from healthcare professionals with expertise in the field

Telemedicine is executed via 3 mediums: video with audio, telephone only, and electronic communication only.

Telemedicine is the delivery of health care services using information or communication technology.

Telemedicine is the use of electronic, digital, internet-based, or telephone-based communication for direct patient care.

Telemedicine platforms = virtual neurological examination

Telemedicine refers to the use of technology to remotely deliver clinical care; the national academy of medicine defines telemedicine as “the use of electronic information and communications technologies to provide and support health care when distance separates the participants;”; “telehealth” broadly as “technology-enabled health and care management and delivery systems that extend capacity and access;” they define this term as comprising a wide range of fields including “ai [artificial intelligence], virtual reality, and behavioral economics.”; however it is defined, telemedicine generally refers to health information that is either synchronously or asynchronously transmitted, or remotely collected.

Telemedicine, or telehealth, is the distribution of information and services via telecommunication and electronic information technologies. this approach to medicine enables long-distance patient and clinician contact, reminders, education, care, intervention, monitoring, and remote admissions

Telemedicine, telehealth or virtual visit is a video visit to a health care provider over a secure network. two-tablet approach for icu and covid-19 affected patients to reduce the healthcare workers’ contact. one digital device is given to the patient and the other to healthcare personnel.

Telemedicine, the use of electronic information and communication technologies to provide health care

Telemedicine, which includes office visits and other medical services provided at a distance using interactive two‐way telecommunications systems (i.e., real‐time audio and video),

Telemedicine, which literally means “healing at a distance; remote care approaches (i.e. telephone- and video-based consultations)

Telemedicine: real-time audio-visual interaction between patient and provider

Telemedicine: a non-public-facing remote communication product allows only the intended parties to communicate

Telemedicine: addresses the diagnosis, treatment and monitoring of patients (including history taking and appropriate physical examination) by means of electronic technology.

Telemedicine: clinically managed effectively from a distance

Telemedicine: phone and ulceration pictures

Telemedicine: synchronous live consultation via video

Telemedicine: teaching techniques based on telemedicine, referred as “tele-education”

Telemedicine: Two-way, live communication between the patient and provider at a distant site including audio and video equipment

Telemedicine: use of audio-visual technology in the provision of medical care remotely

Telemedicine: use of telecommunication and information technologies to provide clinical health care to distant or isolated individuals. using such technology, clinicians can examine patients and make treatment recommendations across long distances

Telemedicine: uses electronic and digital platforms to exchange medical information for improved consumer health

Telemedicine: various audio-visual, telephonic, and internet-based mediums

Telemedicine= provision of health care services remotely

Telephone calls, and instant messaging, including email, health system messaging services, and mobile apps to track patient recorded telephone service available to healthcare providers working in a hospital

Telepsychiatry, or the use of technology to provide mental health services

Telepsychiatry: the delivery of psychiatric care remotely through telecommunications technology

Telerehabilitation: (providing information and advice to persons with disabling conditions)

Telerehabilitation: aims to provide rehabilitation services to individuals in remote locations. used videoconferencing (zoom by zoom video communications inc, San Jose, CA) and remote-control software

Telerehabilitation represents an emerging and innovative approach that can constitute a valid support during the home rehabilitation process for the improvement of motor, cognitive, or psychological disorders.

Teleteaching: live teleteaching video conference platforms whereby student engagement and interactivity can be preserved

The World Health Organization broadly defines telemedicine as the delivery of health care services by all health care professionals using technology for the exchange of valid information for the diagnosis, treatment, and prevention of disease and injuries.4 as an alternative to the traditional office encounter, telemedicine refers to a live (synchronous), two‐way, interactive audio and video‐based communication between the patient and the clinician to deliver care at a distance.

Therapist–guided synchronous (ie, live and interactive) telehealth exercise program: all exercise sessions were delivered via a telehealth platform on a tablet computer.

Video consultations: ‘real-time interactions between patients and/or relatives and health care professionals that take place via video, thereby allowing participants to have audio/visual contact"

Virtual care: any form of health care delivered without the patient and the clinician being present in the same physical location telehealth: the various digital communication modalities and applications that empower care to be delivered irrespective of space. this includes remote monitoring, store-and-forward technology, mobile health applications, and direct patient care.

Virtual check-in: a brief visit between patient and provider using telephone or teleconferencing software to determine whether in-person evaluation is needed.

Virtual check-ins: brief communication between the provider and an established patient via telephone, audio visual application, secure text messaging, e-mail, or a patient portal.

Virtual fracture clinics (vfcs) is a multidisciplinary set-up and decision-making involving the clinician, physiotherapist or advance nurse practitioner to provide therapy guidance and administrative support to type letters. in the field of trauma and orthopedics,

Virtual reality: developing simulated expertise which is somewhat similar to the real-time situation.

Virtual support: clinical and community supports involving broadly increased in-home acute and primary care
